# Comparison analysis between standard polysomnographic data and in-ear-electroencephalography signals: a preliminary study

**DOI:** 10.1093/sleepadvances/zpae087

**Published:** 2024-11-29

**Authors:** Gianpaolo Palo, Luigi Fiorillo, Giuliana Monachino, Michal Bechny, Michel Wälti, Elias Meier, Francesca Pentimalli Biscaretti di Ruffia, Mark Melnykowycz, Athina Tzovara, Valentina Agostini, Francesca Dalia Faraci

**Affiliations:** Department of Innovative Technologies, Institute of Digital Technologies for Personalized Healthcare (MeDiTech), University of Applied Sciences and Arts of Southern Switzerland, Lugano, Switzerland; Department of Electronics and Telecommunications, Politecnico di Torino, Torino, Italy; Department of Innovative Technologies, Institute of Digital Technologies for Personalized Healthcare (MeDiTech), University of Applied Sciences and Arts of Southern Switzerland, Lugano, Switzerland; Department of Innovative Technologies, Institute of Digital Technologies for Personalized Healthcare (MeDiTech), University of Applied Sciences and Arts of Southern Switzerland, Lugano, Switzerland; Institute of Computer Science, University of Bern, Bern, Switzerland; Department of Innovative Technologies, Institute of Digital Technologies for Personalized Healthcare (MeDiTech), University of Applied Sciences and Arts of Southern Switzerland, Lugano, Switzerland; Institute of Computer Science, University of Bern, Bern, Switzerland; IDUN Technologies AG, Glattpark, Switzerland; IDUN Technologies AG, Glattpark, Switzerland; IDUN Technologies AG, Glattpark, Switzerland; IDUN Technologies AG, Glattpark, Switzerland; Institute of Computer Science, University of Bern, Bern, Switzerland; Department of Electronics and Telecommunications, Politecnico di Torino, Torino, Italy; Department of Innovative Technologies, Institute of Digital Technologies for Personalized Healthcare (MeDiTech), University of Applied Sciences and Arts of Southern Switzerland, Lugano, Switzerland

**Keywords:** sleep wearables, in-ear-EEG, machine learning, sleep staging, multisource-scored sleep databases

## Abstract

**Study Objectives:**

Polysomnography (PSG) currently serves as the benchmark for evaluating sleep disorders. Its discomfort makes long-term monitoring unfeasible, leading to bias in sleep quality assessment. Hence, less invasive, cost-effective, and portable alternatives need to be explored. One promising contender is the in-ear-electroencephalography (EEG) sensor. This study aims to establish a methodology to assess the similarity between the single-channel in-ear-EEG and standard PSG derivations.

**Methods:**

The study involves 4-hour signals recorded from 10 healthy subjects aged 18–60 years. Recordings are analyzed following two complementary approaches: (1) a hypnogram-based analysis aimed at assessing the agreement between PSG and in-ear-EEG-derived hypnograms; and (2) a feature- and analysis-based on time- and frequency-domain feature extraction, unsupervised feature selection, and definition of Feature-based Similarity Index via Jensen–Shannon Divergence (JSD-FSI).

**Results:**

We find large variability between PSG and in-ear-EEG hypnograms scored by the same sleep expert according to Cohen’s kappa metric, with significantly greater agreements for PSG scorers than for in-ear-EEG scorers (*p* < .001) based on Fleiss’ kappa metric. On average, we demonstrate a high similarity between PSG and in-ear-EEG signals in terms of JSD-FSI—0.79 ± 0.06—awake, 0.77 ± 0.07—nonrapid eye movement, and 0.67 ± 0.10—rapid eye movement—and in line with the similarity values computed independently on standard PSG channel combinations.

**Conclusions:**

In-ear-EEG is a valuable solution for home-based sleep monitoring; however, further studies with a larger and more heterogeneous dataset are needed.

**Statement of Significance**

Traditional polysomnography (PSG) may prevent from depicting real sleep patterns due to the extensive setting employed. An alternative to overcome this limitation is to use wearable solutions like in-ear-electroencephalography (EEG). To date, the in-ear-EEG and the standard PSG derivations have only been compared following basic correlation analysis. We propose a more exhaustive methodology—hypnogram- and feature-based—to evaluate the similarity between the in-ear-EEG and PSG signals. The ultimate goal is to investigate whether in-ear-EEG sensors inherit information close to the ones we extract through standard PSG.

## Introduction

Sleep is essential to good health [1]. Poor or inadequate sleep is associated with several dysfunctions in most physiological systems [2]. Sleep analysis is of crucial importance in the diagnosis and treatment of sleep disorders [1, 2].

Polysomnography (PSG) is the gold standard to perform sleep studies [1–3]. PSG is performed in appropriate clinical facilities and involves recording multiple bio-signals during a full night’s sleep, including brain activity (electroencephalography [EEG]), eye movements (electrooculography [EOG]), muscle activity (electromyography [EMG]), cardiac activity (electrocardiography [ECG]), body position, breathing effort, blood saturation, etc. [1, 2]. The PSG recordings are nowadays manually evaluated by trained personnel according to the American Academy of Sleep Medicine (AASM) manual [4]. Despite being highly standardized by AASM guidelines, this manual procedure is time- and effort-consuming, and it is not error-free [5]. These limitations, along with the saturation of the sleep units, lead to high costs related to patient management and care. Besides, due to the invasive equipment, and since the patients typically sleep in an atypical and unfamiliar environment, standard PSG-based analyses introduce biases to the sleep quality assessment [1, 2].

Wearable and portable devices may be valid solutions, as they allow for home-based sleep monitoring. The use of unconventional channels has been widely explored in the field of mobile sleep monitoring with wearable devices. A comprehensive overview of sensing technologies (different signals and their combinations) for sleep staging via wearable devices is provided in [6]. The signals conveying a substantial amount of information for this task are EEG, EOG, and EMG—with the EEG signal being the most used sensing modality as a single data source. We might therefore speculate that ear-EEG may be the right choice. The brain activity is recorded from electrodes placed in or around the ear, while also leading to several advantages in comfort, fixed electrode positions, robustness to electromagnetic interference, and ease of use [1, 7].

To date, only two research groups [2, 8–13] have exploited ear-EEG signals for sleep analysis. The majority of their studies mainly relied on feature-based methodologies to evaluate the feasibility of ear-EEG technology for automated sleep monitoring. They showed that automatic sleep scoring based on ear-EEG signals was performing at levels comparable to expert scoring of PSG, in young healthy subjects [2, 8–13].

Jørgensen et al. [14] study can be seen as a proof of concept for the suitability of ear-EEG on epileptic subjects and in [15], Kjaer et al. showed that sleep metrics computed from multiple nights automatically scored on ear-EEG are more reliable than the ones computed from a single night manually scored via standard PSG. Thus, highlighting how ear-EEG seems to be a useful alternative for sleep staging for the single night recording and an advantageous choice for several nights of sleep monitoring.

However, in none of the above-mentioned studies—even before inferring and/or validating sleep metrics and/or algorithms on these promising signals—the similarity between each standard PSG and the ear-EEG derivations has been thoroughly investigated or quantified.

In this work, we carried out the above-mentioned comparison analysis, first focusing on the sleep scoring procedure (*hypnogram-based*), and then directly evaluating the signals (*feature-based*). In the *Methods* section, we briefly describe the dataset along with the instrumentation, data collection procedure, and preprocessing of the signals. In the *Comparison analysis: hypnogram-based approach* subsection, we first describe how to define the consensus in a multisource-scored dataset. The hypnogram-based comparison analysis is performed by evaluating the intra- and interscorer variability, thus assessing the agreement (i.e. Cohen’s kappa and Fleiss’ kappa) between the PSG and in-ear-EEG-derived hypnograms. In the *Comparison analysis: feature-based approach* subsection, we present all the steps of our feature-based comparison analysis, that is, time- and frequency-domain feature extraction, feature selection, and the final evaluation of the similarity between the two different sources. The proposed approach relies on a comparison of the distributions of the selected features—extracted from the two different sources, PSG and in-ear-EEG, respectively—via the newly introduced Jensen–Shannon Divergence Feature-based Similarity Index (JSD-FSI). In-ear-EEG earbuds, sensors are thought to perform better—for sleep-scoring tasks—when combined with additional EOG signals [3, 10, 16]–especially in distinguishing the rapid eye movement (REM) sleep stage. Therefore, we extract features from both EOG and scalp-EEG derivations (i.e. frontal, central, and occipital brain regions), and we compare them to the features extracted from the in-ear-EEG recordings. Among the scalp-EEG derivations, we also included the mastoid-to-mastoid one (M1-M2), as its information has been proven to be similar to the in-ear-EEG [7, 11].

In the *Results* section, we present the most significant outcomes of our hypnogram- and feature-based approaches, validating any related observations through appropriate statistical analyses. Finally, in the *Conclusions* section, we discuss the main key points and implications of our findings, highlighting the contributions of the current study as well as the limitations encountered.

To summarize, in this work, we investigate whether or not in-ear-EEG sensors inherit information—or set of features—close to what we usually extrapolate through standard PSG derivations. The primary research questions are the following:

1. How similar are hypnograms derived from PSG versus in-ear EEG signals, specifically in terms of their scoring procedures?
2. How similar are the PSG and in-ear EEG signals across different sleep stages?
3. Does the similarity vary among different sleep stages?
4. Can in-ear EEG sensors provide information comparable to that obtained from a standard PSG setup?

## Methods

We exploit an already existing dataset collected during an observational study carried out at IDUN Technologies. In the hypnogram-based comparison analysis, we first describe how to compute the consensus in the multisource-scored dataset (i.e. a dataset where each recording is scored by multiple experts and looking at different sources of signals), and then we assess the intra- and interscorer variability. Establishing a consensus is crucial to better conduct the feature-based comparison analysis—that is, analyzing only the sleep epochs where the PSG-scorers and the in-ear-EEG-scorers were in agreement on the associated sleep period.

Indeed, in our feature-based comparison procedure, we evaluate the similarity between the signals coming from two different sources for each sleep stage independently. The feature-based analysis is divided into three steps: feature extraction (time- and frequency-domain features), feature selection, and calculation of the newly defined JSD-FSI.

### Dataset

**Figure 1.**
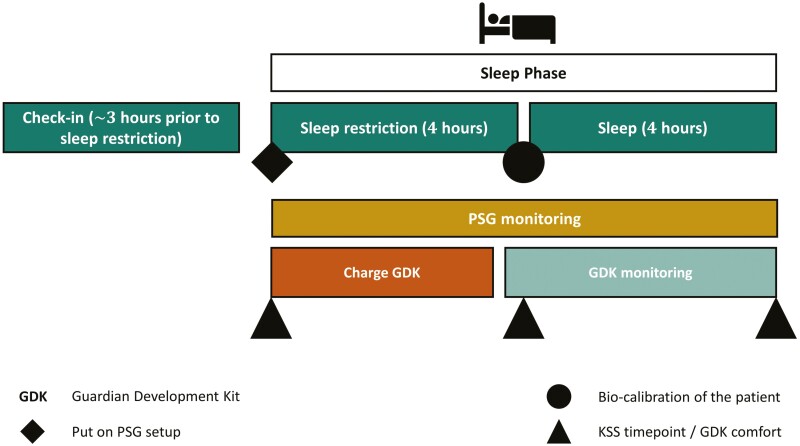
Schematic layout of the quality assurance study and the data collection procedure.

The quality assurance study (BASEC Nr. Req-2022-00105) involves 10 healthy subjects, including both females and males (18–60 years) selected according to the Pittsburgh Sleep Quality Index [17]. Following a screening period of 28 days, the subjects experience one overnight stay at the investigational site. Participants are monitored using multiple standard surface electrodes on their scalp (EEG), outer canthus of each eye (EOG), mentalis (chin), torso (EKG) in the conventional PSG monitoring, and an additional in-ear-EEG sensing technology monitoring. Participants arrive ~3 hours prior to their normal bedtime at the sleep laboratory, and they are instructed about the overall study, including PSG preparation/setting phase, a sleep restriction phase ≈4 hours, and the sleep phase ≈4 hours (Figure 1).

The subjects are asked to avoid caffeine intake from noon before arriving at the study center. They perform specific head and eye movements for biocalibrating the instrumentation used for data collection. Ear tips of three different sizes are given to participants to provide proper fit and electrical contact. A 10-minute stabilization period is awaited before starting data collection to ensure the in-ear electrodes reach thermal equilibrium with the participant’s body temperature. For each participant, an impedance measurement of the in-ear-EEG signal is performed at 31.2 Hz to guarantee that the system is stable. A 300 kΩ threshold is set for good signal quality—greater values indicate either electrical malfunctions or incorrect placement of the ear tips in the ear.

The sleep restriction starts at the in-bed time of each subject and lasts for about 4 hours, after which participants are allowed to sleep for another 4 hours. During the last hour of the sleep restriction phase, the subjects abstain from using electronic devices. The sleep room is set up according to AASM guidelines [4]. The Karolinska Sleepiness Scale [18] is administered at the beginning and the end of the sleep restriction period, and prior to sleep to assess subjective drowsiness.

PSG and in-ear-EEG signals are recorded simultaneously. The data analyzed in this study refer only to the 4 hours recorded during sleep.

**Figure 2.**
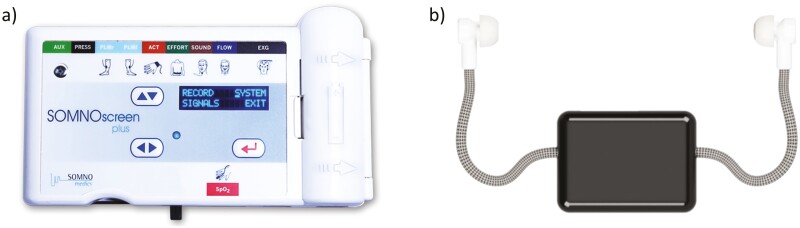
Devices employed in the data collection: (A) SOMNOmedics SOMNOscreen plus system for PSG data with the EXG configuration, that is, including six scalp electrodes, EOG, and ECG signal monitoring; (B) GDK hardware including ear tips, earpieces, and brain box used to record in-ear-EEG data.

PSG signals are collected using a SOMNOmedics SOMNOscreen plus system with a sampling frequency of 256 Hz (Figure 2A). The signals are band-pass filtered between 0.2 and 35 Hz and ECG artifacts are removed automatically by the recording device. A total of 21 channels are investigated, considering both bipolar and unipolar derivations: two reference electrodes (M1 and M2); six EEG bipolar derivations (C3-M2, F3-M2, O1-M2, C4-M1, F4-M1, and O2-M1); six EEG unipolar derivations (C3, C4, F3, F4, O1, and O2); four EOG bipolar derivations (E1-M1, E1-M2, E2-M1, and E2-M2); two EOG unipolar derivations (E1, and E2); the mastoid-to-mastoid derivation (M2-M1). From here on, we will refer to the set of PSG channels as the set *Q*.

In-ear-EEG signals are collected via the Guardian Development Kit (GDK) device designed by IDUN Technology with a sampling frequency of 250 Hz (Figure 2B). The GDK system includes hardware (Brain Computer Interface) and streaming software (Neuro-Intelligence Platform). The former involves two dry contact electrodes designed by IDUN Technology, that is, Dryode Ink electrodes, which are made of an elastomer material functionalized by an electrically conductive coating. The recording and reference channels are placed in the right and left ears, respectively. Biopotential differences between electrodes are measured using the ADS1299-x amplifier (Texas Instruments, LLC, Dallas, TX).

The in-ear-EEG signals are band-pass filtered between 0.5 and 35 Hz, before being normalized as their amplitude range would match the one of the simultaneously recorded PSG signals. In-ear-EEG recordings are multiplied with the standard deviation ratio of PSG and in-ear-EEG data. From here on, we will refer to the single in-ear-EEG channel as CH1 channel. PSG and in-ear-EEG signals are manually synchronized based on easily distinguishable artifacts in both data streams. They are then trimmed such that the recordings referring to the same subject share the same length.

In Supplementary Figures 1 and 2, for each subject, we report an example of a 30-second epoch of raw and preprocessed in-ear-EEG signal, respectively.

Three scoring experts have independently scored both signals—first evaluating PSG and then in-ear-EEG data, according to AASM guidelines [4]. This results in three PSG hypnograms and three in-ear-EEG hypnograms, for each subject. The dataset contains the following annotations W, N1, N2, N3, REM, MOVEMENT, and UNKNOWN, where the last two refer, respectively, to movement artifacts and to no sleep stage assigned. In this study, the three non-REM (NREM) sleep stages are combined together under the label NREM, and all the epochs scored as MOVEMENT or UNKNOWN are not considered.

### Comparison analysis: hypnogram-based approach

*Consensus in a multisource-scored dataset*. In the hypnogram-based approach, we first compute the consensus among the three scorers on each data source—inspired by previous studies [19, 20] analyzing multiscored databases. The majority vote from the scorers has been computed—that is, we assign to each 30-second epoch the most voted sleep stage among the scorers. In the case of ties, we compute the soft-agreement metric [20] to then consider the label from the most reliable scorer. The most reliable scorer is the one that is frequently in agreement with all the others. We then rank the reliability of each scorer, to finally define the most reliable scorer, for each subject.

We denote with *J* the total number of scorers, with *j* the scorer for which the soft-agreement metric is evaluated, and with *i* all the other scorers. The one-hot encoded sleep stages given by the scorer *j* are ${\hat{y}}_{j}\;\in {\left[0,1\right]}^{KxT}$, that is, 1 assigned for the scored stage and 0 for the other stages, *K* is the number of classes, that is, *K* = 3 sleep stages, and *T* is the total number of epochs. The probabilistic consensus ${\hat{z}}_{j}$ among the *J* − 1 scorers (*j* epochs as follows:


$$\begin{array}{l} {\hat{z}}_{j}=\frac{\overset{J}{\underset{i=1}{\sum }}\;{\hat{y}}_{i}\left[t\right]}{max\;\overset{J}{\underset{i=1}{\sum }}\;{\hat{y}}_{i}\left[t\right]}\;\forall t;\;i\neq j \end{array}$$ (1)

where *t* is the *t*th epoch of *T* epochs and ${\hat{z}}_{j}\in {\left[0,1\right]}^{KxT}$, that is, 1 is assigned to a stage if it matches the majority or if it is involved in a tie. The maximum function in the denominator is used to combine the contributions from multiple scorers while ensuring that the scale of values remains within the range [0, 1]. The Soft−Agreement is then computed separately for each scorer across all the *T* epochs as follows:


$$\begin{array}{l} Soft-Agreement_{j}=\;\frac{1}{T}\;\overset{T}{\underset{t=0}{\sum }}{\hat{z}}_{j}\left[y_{j}\right] \end{array}$$ (2)

where ${\hat{z}}_{j}\left[y_{j}\right]$ denotes the probabilistic consensus of the sleep stage chosen by the scorer *j* for the *t*th epoch. Soft-$Agreement_{j}\in \;\left[0,1\right]$, where the 0 value is assigned if the scorer *j* systematically scores all the annotations incorrectly compared to the others, while 1 is assigned if the scorer *j* is always involved in tie cases or in the majority vote. The Soft-Agreement is computed for all the scorers, and the values are sorted from the highest—high reliability—to the lowest—low reliability.

The Soft-Agreement is computed for each subject, that is, the scorers are ranked accordingly, and in case of a tie the top-1 scorer will be the one used for that subject. In Supplementary Tables 1 and 2, we report the Soft-Agreement values computed on each of the three scorers, and for each subject, on the PSG and in-ear-EEG data sources respectively.

Intra- and interscorer variability are then assessed using Cohen’s kappa and Fleiss’ kappa metrics [21], respectively. The intrascorer variability refers to the comparison between PSG and in-ear-EEG hypnograms scored by the same clinician, while the interscorer variability characterizes the agreement among scorers related to the same source, that is, either PSG or in-ear-EEG scorers. According to Landis and Koch [14], Cohen’s kappa values exceeding 0.80 suggests an almost-perfect agreement between scorers; a range of 0.61–0.80 indicates substantial agreement, whereas 0.41–0.60 implies moderate agreement. Fair agreement falls in the range of 0.21–0.40, and slight agreement occurs between 0.00 and 0.20. Fleiss’ kappa values are interpreted in the same way [21].

In the feature-based comparison analysis, we define similarity-scores between the two different data sources, PSG and in-ear-EEG, exploiting a per-sleep-stage-based approach. Hence, we first define a common label-ground-truth reference for both types of signal—to prevent additional bias in our analysis. Such a reference is defined by all the epochs scored in the same sleep stage by both the consensus, tha tis, PSG and in-ear-EEG scoring procedures. Therefore, for each subject, starting from the PSG and in-ear-EEG hypnograms, we first evaluate the consensus among the three expert scorers for the PSG and the in-ear-EEG, respectively, and then we consider only the epochs where these two consensus are in agreement for our sleep stage-wise feature analysis. In Table 1, we report a summary of the total number and percentage of the epochs per sleep stage for both PSG and in-ear-EEG-based scoring procedure, and the intersection (∩) computed on the labels coming from the two different sources.

**Table 1.** Number and percentage of 30-second epochs per sleep stage (i.e. the result of the consensus reached by the three different scorers) for both PSG and in-ear-EEG-based scoring procedure, and the intersection (∩) computed on the labels coming from the two different sources

	W	NREM	REM	Total
PSG	344 (7.5%)	3469 (75.9%)	755 (16.5%)	4568
In-ear-EEG	277 (6.1%)	3768 (82.5%)	523 (11.4%)	4568
PSG ∩ In-ear-EEG	236 (6.0%)	3308 (84.0%)	392 (10.0%)	3936

**Figure 3.**
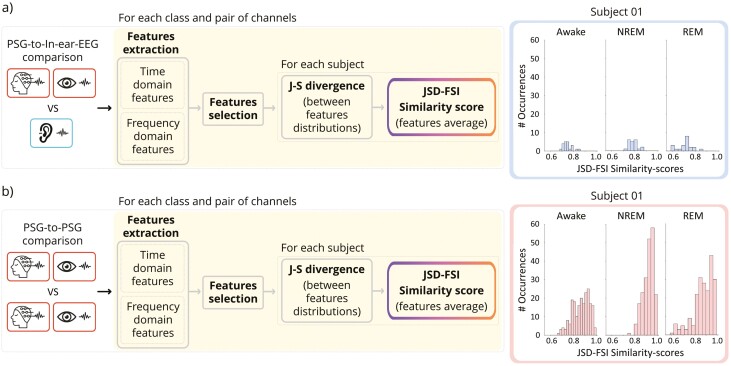
Workflow for evaluating the similarity between the signals recorded from two different channels, including feature extraction and feature selection, separately for each sleep stage; and the comparison between feature distributions using the Jensen–Shannon divergence before the assessment of the similarity-scores, individually for each sleep stage and for each subject. In detail, (A) refers to the comparison between one in-ear-EEG and one PSG channel (either scalp-EEG or EOG channels); while (B) illustrates the analysis between two PSG channels (either scalp-EEG or EOG channels). An example of similarity-scores distribution for awake, NREM, and REM classes is included for both case studies.

*Comparison analysis: feature-based approach*. In order to assess the similarity between standard PSG and in-ear-EEG signals, we follow specific steps (as summarized in Figure 3A): (1) we extract time- and frequency-domain features from the above-mentioned PSG derivations and the in-ear-EEG channel on each 30-second sleep epoch; (2) we remove all redundant features through pairwise assessments (feature selection procedure) to identify those conveying the same information; and (3) we then define the JSD-FSI exploited to compare the distributions of the selected features, for each sleep stage and for each subject on both PSG derivations and the in-ear-EEG.

To fairly validate the results of our comparison analysis, we decided to also quantify the similarity between all the possible combinations of PSG-derived signals, including scalp-EEG and EOG channels (Figure 3B). The idea is to assess that the PSG-to-In-ear-EEG JSD-FSI similarity scores (histograms in blue Figure 3A) are on average close to those we derive from the standard PSG-to-PSG comparisons (histograms in red Figure 3B). The results are evaluated separately for each sleep stage and for each subject.

### Feature extraction

We extract both time- and frequency-domain features from 30-second epochs of signals. All the features depending on the amplitude are computed on signals normalized by their maxima, compensating for differences in magnitudes.

#### Time-domain features.

To better compare two different sources of brain activity, we evaluate the similarity based on the extraction of several features that characterize the EEG signal from multiple perspectives. First, we compute some standard descriptive statistics (i.e. standard deviation, interquartile range, skewness, and kurtosis), the maximum first derivative, and the number of zero-crossings [22]. These features provide insights regarding the distribution of the EEG data and the level of neural activity, that is, depolarization rate. To evaluate the regularity and predictability of EEG signals in terms of consistency and repetition of patterns over time, we then include entropy-based measures, specifically, the approximate entropy [23–25], the sample entropy [23–27], the Singular Value Decomposition entropy [26, 27], and the permutation entropy [26, 28]. To assess the complexity in terms of long-range correlations and self-similarity within the EEG signal, we consider the Lempel-Ziv complexity [29] and the Detrended Fluctuation Analysis (DFA) exponent [30, 31]. The Lempel-Yiv complexity and DFA both rely on the evaluation of recurring sub-segments of signal within the time series [29–31]. In addition, we also include Hjorth parameters of activity, mobility, and complexity [32], and the Katz, Higuchi [33], and Petrosian [34] fractal dimensions [35, 36] to further quantify the complexity or irregularity of the analyzed time series. In detail, while Hjorth parameters offer a quantitative characterization of the morphology of the EEG signal by directly focusing on its amplitude fluctuations [31], fractal dimensions provide insights into its self-similarity across multiple temporal scales [32–35].

#### Frequency-domain features.

We first compute the power spectral density (PSD) of each 30-second signal using Welch’s average periodogram method [37]. We choose a Hamming window of 5-second length with a 50% overlap, resulting in a frequency resolution of 0.2 Hz [31, 38]. We exploited the Hamming window to reduce the estimation variance, the side-lobe effect, and the spectral leakage phenomena [39]. The 5-second window length is set to be at least twice the lowest frequency of interest 0.5 Hz (i.e. the lower end of the EEG delta power band) [38]. Then, the median-average partially mitigates the influence of any noise/artifacts we have on our signals [31, 38].

Once the PSD has been computed, we first extract standard frequency-domain features, such as the spectral energy of the whole 30-second signal, and the relative spectral power on all the EEG frequency bands, that is,. delta (δ, 0.5–4 Hz), theta (θ, 4–8 Hz), alpha (α, 8–12 Hz), sigma (σ, 12–16 Hz), beta (β, 16–30 Hz), and gamma (γ, 30–35 Hz). We then include several ratio measures between the different frequency bands, that is, δ/θ, δ/σ, δ/β, θ/α, δ/α, α/β, δ/(α + β), θ/(α + β), and δ/(α + β + θ).

In our frequency-domain analysis, we include additional features assessing the spread, symmetry, tail behavior, shape, and complexity of each 30-second signal’s spectrum distribution. Specifically, we compute the four central moments in statistics (i.e. mean, variance, skewness, and kurtosis), the spectral entropy and the Renyi entropy [40], the spectral centroid [41, 42], the spectral crest factor [43], the spectral flatness [41, 42], the spectral roll-off [44], and the spectral spread [42].

In Supplementary Tables 3 and 4, we report the complete list of all the time- and frequency-domain features extracted. In the *Supplementary Analyses* section, we also include additional mathematical details for each of the above features.

#### Feature selection.

The feature selection procedure is essential to remove in our analysis possible redundancy within the feature subset. We exploit a feature selection algorithm based on pairwise feature correlation [45, 46], aiming to identify the most representative features among all the extracted ones. This algorithm is selected because it outperforms other traditional feature selection methods on several real-life datasets [44]. Additionally, an unsupervised approach is used since there are no labels related to the similarity between PSG and in-ear-EEG signals. However, before proceeding with this procedure, we should first consider that the above-derived features are meant to describe the morphology of our neurological signals. The features are all supposed to change based on the state brain subjects are in, that is, waking state, NREM state, or REM state. Thus, we decide to first divide the data, that is, the 30-second epochs, depending on the sleep stage they are assigned to.

Therefore, for each pair of channels (i.e. a pair is defined as, where q∈Q, and *Q* is the above-defined set of PSG channels), we build pairs of datasets $\left\{D^{q},\;D^{CH1}\right\}$, one pair for each of the k∈K sleep stages. Each dataset $D\in {ℜ}^{MxNxK}$ is the result of concatenated feature vectors ${\underline{\;f}}_{n,\;k}$, where *K* = 3 is the number of sleep stages, *N* is the total number of features, with n∈N, and *M* is the total number of 30-second epochs in each stage.

A *z*-score normalization is performed separately on each dataset-pair, $D^{q}$ and $D^{CH1}$, and for each sleep stage, to reduce dissimilarities among the different subjects. On each dataset, we perform the feature selection based on the computation of a modified version of the maximal information compression index between each pair of features [45–47]. As the algorithm adopts a *k*-nearest neighbors approach, the determination of the initial *k* value is crucial and is guided by metrics such as the representation entropy [45, 46] and the redundancy rate [46, 48].

*In Supplementary Analyses* section, we report additional mathematical details regarding the modified version of the feature selection algorithm, along with further details on the *k*-nearest neighbors approach.

### Jensen–Shannon Divergence–Feature-Based Similarity Index

We quantify the similarity between pairs of feature distributions, coming from two different sources (e.g. PSG-derived and in-ear-EEG-derived), exploiting the JSD [49]. The JSD divergence is a symmetric and smoothed version of the Kullback–Leibler (KL) divergence [50], quantifying the similarity between two probability distributions. Practically, we first compute the probability density function (PDF) Φ for each pair of feature distributions $\left\{\Phi \left({\underset{-}{f}}_{n,\;k}^{\;q}\right),\;\Phi \left(\;{\underset{-}{f}}_{n,\;k}^{\;CH1}\right)\right\}$ extracted from the paired datasets $\left\{D^{q},\;D^{CH1}\right\}$ [51]. We then measure the dissimilarities between each pair via the JSD divergence. JSD ranges from 0 (identical distributions) to 1 (completely dissimilar distributions). The higher the number, the more dissimilar the probability distributions. Hence, once we compute the JSD metric on each PDF feature pair $\left\{\Phi \left({\underset{-}{f}}_{n,\;k}^{\;q}\right),\;\Phi \left(\;{\underset{-}{f}}_{n,\;k}^{\;CH1}\right)\right\}$, for each sleep stage *k* and of each subject, we can finally compute the JSD-FSI between each PSG *q* channel and the in-ear-EEG *CH*1 channel as follows:


$$\begin{array}{l} JSD\text{-}FSI_{k\;\in \;K}=\overset{N}{\underset{n=1}{\sum }}\left(1-JSD_{n}\right)\;/\;N\; \end{array}$$ (3)

$$\begin{array}{l} JSD_{n}\left(\Phi \left({\underline{f}}_{n,\;k}^{\;q}\right)||\Phi \left({\underline{f}}_{n,\;k}^{\;CH1}\right)\right)=\left(KL\left(\Phi \left({\underline{f}}_{n,\;k}^{\;q}\right)||M\right)KL\left(\Phi \left({\underline{f}}_{n,\;k}^{\;CH1}\right)||M\right)\right)/2 \end{array}$$ (4)

where *N* is the total number of features, *M* is the *average* distribution defined as $M=\left(\Phi \left({\underline{\;f}}_{n,\;k}^{\;q}\right)+\Phi ({\underline{\;f}}_{n,\;k}^{\;CH1}\right)/2$, while $KL\left(\Phi \left({\underline{f}}_{n,\;k}^{\;q}\right)\;||\;M\right)$ is the KL divergence between the two distributions $\Phi \left({\underline{f}}_{n,\;k}^{\;q}\right)$ and *M*_,_ defined as


$$\begin{array}{l} KL(\Phi \left({\underset{-}{f}}_{n,\;k}^{\;q}\right)\;||\;M)=\overset{}{\underset{}{\sum }}f_{k}^{\;q}\left(t\right)\times log\left(\;f_{k}^{\;q}\left(t\right)\;/\;M\left(t\right)\right)\;\;\forall t; \end{array}$$ (5)

Hence, for each sleep stage and for each subject, we derive 21 (i.e. total number of pairs comparisons {q, CH1}) JSD-FSI similarity scores. Each of these scores is defined as the sum of the individual $JSD_{n}$ similarity scores—resulting from all the PDF feature distribution comparisons—divided by the total number of features analyzed (Equation 2).

The same comparison analysis has been done between all the possible combinations of PSG-derived signals, that is, scalp-EEG-to-scalp-EEG, scalp-EEG-to-EOG, and EOG-to-EOG comparisons. We compare all the PDF feature distributions extracted from all the channels in the PSG set *Q* (unique comparisons, i.e. upper triangle of the symmetric matrix with dimension (|Q| x|Q|), where |Q|=21 is the cardinality of the set, or the total number of PSG channels). Hence, we first compute the PDF Φ for each pair of feature distributions $\left\{\Phi \left({\underset{-}{f}}_{n,\;k}^{\;q_{i}}\right),\;\Phi \left(\;{\underset{-}{f}}_{n,\;k}^{\;q_{j}}\right)\right\}$ extracted from the dataset pairs $\left\{D^{q_{i}},\;D^{q_{j}}\right\}$, with  i≠j. We then compute the JSD-FSI similarity-scores on all the possible combinations as described above. In that case, for each sleep stage and subject, we derive 210 (i.e. unique comparisons between all the PSG channels via the binomial coefficient $\frac{\left|Q\right|!}{2!\left(\left|Q\right|-2\right)!}$) JSD-FSI similarity-scores.

We will fairly assess, for each sleep stage and subject, that the PSG-to-in-ear-EEG JSD-FSI similarity scores are on average close to those derived from the standard PSG-to-PSG comparisons.

## Results

The main contributions of our study are the following:

1. We found large intrascorer variability related to the in-ear-EEG scoring compared to the PSG scoring, with agreements among PSG scorers significantly (*p* < .001) greater than the ones among in-ear-EEG scorers. This difference is probably due to the uncertainty the scorers have when evaluating in-ear-EEG signals.
2. We show that the similarity between the PSG and the in-ear-EEG signals—in terms of JSD-FSI score—is high, on average 0.79 ± 0.06 in awake, 0.77 ± 0.07 in NREM, and 0.67 ± 0.10 in REM.
3. We found significant changes in JSD-FSI scores between sleep stages, with significantly greater values for the awake stage with respect to NREM (*p* < .001) and REM (*p* < .001) stages, and significantly greater values for the NREM stage compared to REM (*p* < .001) stage.
4. We prove that the JSD-FSI similarity values reported in (Equation 2) are revealed to be in line/overlap with the similarity values computed independently on the different combinations of PSG channels.

### Comparison analysis: hypnogram-based approach

#### Intra- and interscorer variability in the multisource-scored dataset.

We measure the agreement between each pair of PSG and in-ear-EEG hypnograms referring to the same recording/subject scored by the same scorer expert, according to Cohen’s kappa and Fleiss’ kappa metrics.

**Figure 4.**
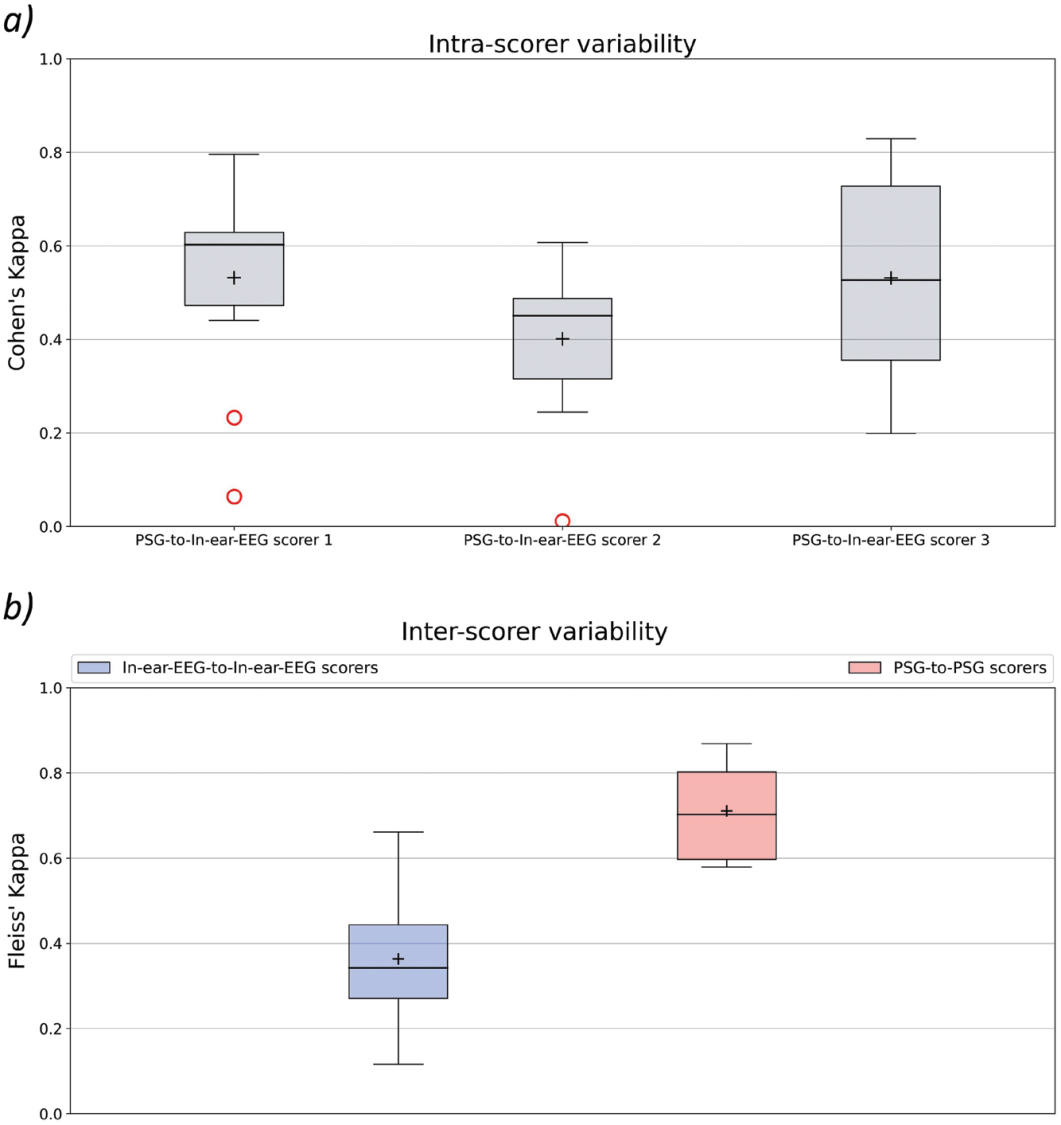
(A) Intrascorer variability (multisource-scored dataset). Boxplot distribution of the Cohen’s kappa values computed for each recording/subject between the PSG and in-ear-EEG hypnograms—for each scorer. (B) Interscorer variability (multisource-scored dataset). Boxplot distributions of the Fleiss’ kappa values computed for each recording/subject between the three scorer experts for in-ear-EEG (in blue) and PSG (in red) signals.

In Figure 4A, we report, for each scorer, the distribution of Cohen’s kappa values computed for each recording/subject between the PSG and in-ear-EEG hypnograms—so quantifying the intrascorer variability in the multisource-scored dataset. In this context, the values exhibit considerable dispersion across all distributions, with limited agreement levels, particularly in the comparison performed on the scorer 2. No significant changes are found among the three Cohen’s kappa distributions according to the ANOVA test (α = 0.05). The normality assumption is verified based on the Shapiro–Wilk test (α = 0.05).

In Figure 4B, we report, for each data source, the distribution of the Fleiss’ kappa values, comparing hypnograms from the same recording/subject, scored by the three expert scorers, first on PSG and then on in-ear-EEG signals—so quantifying the inter-scorer variability in the multisource-scored dataset.

Notably, the three PSG scorers exhibit greater coherence scoring the PSG recordings, compared to when scoring the in-ear-EEG signals. Indeed, Fleiss’ kappa values for PSG hypnograms are found to be significantly greater (*p* < .001) than those between in-ear-EEG hypnograms, based on the Student’s *t*-test (α = 0.05). We employ a parametric statistical test given normal distributions, as stated by the Shapiro–Wilk test (α = 0.05).

For each sleep stage, we also assess the average agreement across all subjects between the PSG and in-ear-EEG consensus (Table 2), finding large discrepancies for the REM stage. The agreement is evaluated according to precision, recall, and F1-score metrics, using the PSG consensus as the gold standard.

**Table 2.** Average precision, recall, and F1-score metrics evaluated across all subjects between PSG and in-ear-EEG consensus, considering the former as the gold standard

	W	NREM	REM
Precision	0.84 ± 0.16	0.88 ± 0.05	0.65 ± 0.35
Recall	0.80 ± 0.21	0.95 ± 0.03	0.47 ± 0.27
F1-score	0.79 ± 0.14	0.91 ± 0.03	0.53 ± 0.28

### Comparison analysis: feature-based

#### JSD-FSI similarity-scores.

**Figure 5.**
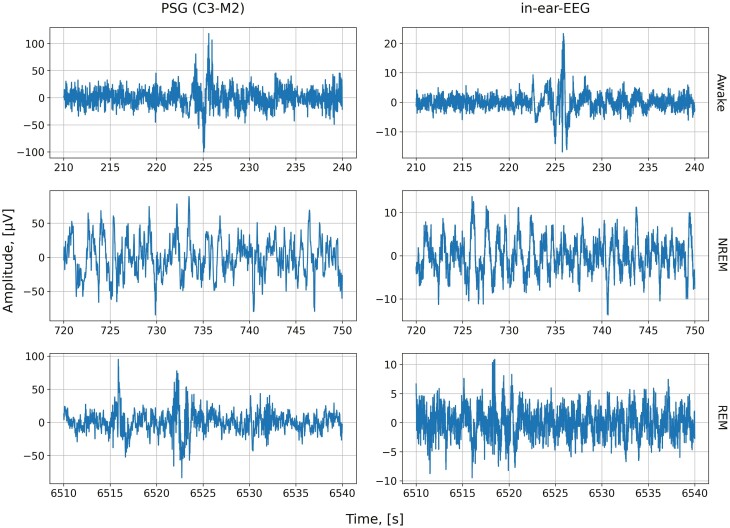
Example of 30-second data samples from both PSG and in-ear EEG recordings, for each sleep stage. The PSG derivation considered is the C3-M2 channel.

For each sleep stage, we report in Figure 5 a representative 30-second epoch of preprocessed PSG and in-ear EEG recordings. These epochs serve as the basis for extracting both temporal and spectral features.

The most frequently selected features across the different datasets $\left\{D^{q},\;D^{CH1}\right\}$ for all the three sleep stages (Supplementary Figure S) are the following: the relative δ, θ, α, and σ power bands; the δ/θ power ratio; the spectral flatness; the spectral variance; the skewness; the kurtosis; the maximum first derivative; and the Hjorth activity and complexity. In addition, there are extra selected features specifically for each sleep stage: the spectral skewness, the interquartile range, the Hjorth mobility, the Renyi entropy, the permutation entropy, and the Higuchi fractal dimension for the awake stage; the relative β and γ power bands, the spectral skewness, the standard deviation, the number of zero-crossings, the spectral entropy and the permutation entropy for the NREM sleep stage; and the spectral energy, the relative γ power band, the spectral kurtosis, the standard deviation, and the spectral entropy for the REM sleep stage. The above-selected features are not to be understood as more or less relevant to the purpose of our comparison analysis. The not-selected features were ignored because of the redundant information they were bringing.

The common label-ground-truth reference (i.e. intersection computed on the labels coming from the two different sources) for subjects 3 and 8 do not show any REM epochs—lack of agreement between corresponding PSG and in-ear-EEG consensus. Therefore, the results will not include JSD-FSI similarity scores for these two subjects in the REM class. Furthermore, whenever the in-ear-EEG channel or any of the PSG channels show some noisy epochs (e.g. no signal—constant amplitude—meaning no reliable indicators of brain activity), we exclude that specific channel. This situation occurs mainly for the channel M2 for subjects 3 and 6—with 79% and 82% of noisy epochs respectively. Hence, we exclude the channel M2 from the analysis for subjects 3 and 6.

In Table 3, we report the averaged JSD-FSI similarity-scores computed between the in-ear-EEG and PSG channels for each subject and for each sleep stage, respectively—in Supplementary Figures 4–6 also as standard topographic images. Overall, the similarity between the PSG and the in-ear-EEG signals—in terms of JSD-FSI score—is high, on average 0.79 ± 0.06 in awake, 0.77 ± 0.07 in NREM, and 0.67 ± 0.10 in REM. On average, there are no substantial differences with the in-ear-EEG compared to the corresponding PSG channel across all subjects. The spatial distributions in terms of JSD-FSI scores (any pair in-ear-EEG and PSG derivations) are on average consistent within the different subjects and channels. According to the Kruskal–Wallis test (α = 0.05), significant changes in JSD-FSI scores are found among sleep stages (*p* < .001). In detail, based on the Mann–Whitney *U*-test (α = 0.05), there is statistical evidence that JSD-FSI similarity scores for the awake stage are greater than NREM (*p* < .001) and REM (*p* < .001) ones; and that JSD-FSI similarity scores for the NREM stage are greater than REM ones (*p* < .001). Nonparametric statistical tests are used as the normality assumption is not met based on the Shapiro–Wilk test (α = 0.05).

**Table 3.** Averaged JSD-FSI similarity scores computed between the in-ear-EEG and PSG channels for each subject and for each sleep stage, respectively

	W	NREM	REM
Subject 01	0.75 ± 0.04	0.79 ± 0.04	0.71 ± 0.06
Subject 02	0.80 ± 0.02	0.80 ± 0.03	0.67 ± 0.05
Subject 03	0.72 ± 0.04	0.75 ± 0.07	-
Subject 04	0.78 ± 0.03	0.82 ± 0.03	0.72 ± 0.04
Subject 05	0.84 ± 0.02	0.82 ± 0.04	0.75 ± 0.03
Subject 06	0.78 ± 0.05	0.73 ± 0.09	0.62 ± 0.05
Subject 07	0.83 ± 0.02	0.80 ± 0.03	0.73 ± 0.04
Subject 08	0.74 ± 0.03	0.67 ± 0.03	–
Subject 09	0.83 ± 0.03	0.73 ± 0.04	0.57 ± 0.05
Subject 10	0.85 ± 0.04	0.79 ± 0.03	0.59 ± 0.06
Average	0.79 ± 0.06	0.77 ± 0.07	0.67 ± 0.10

When assessing the similarity between in-ear-EEG and PSG derivations, and comparing it to the similarity computed among all the possible 210 PSG-to-PSG comparisons (JSD-FSI similarity scores computed from the PSG channels), similar values are observed.

**Figure 6.**
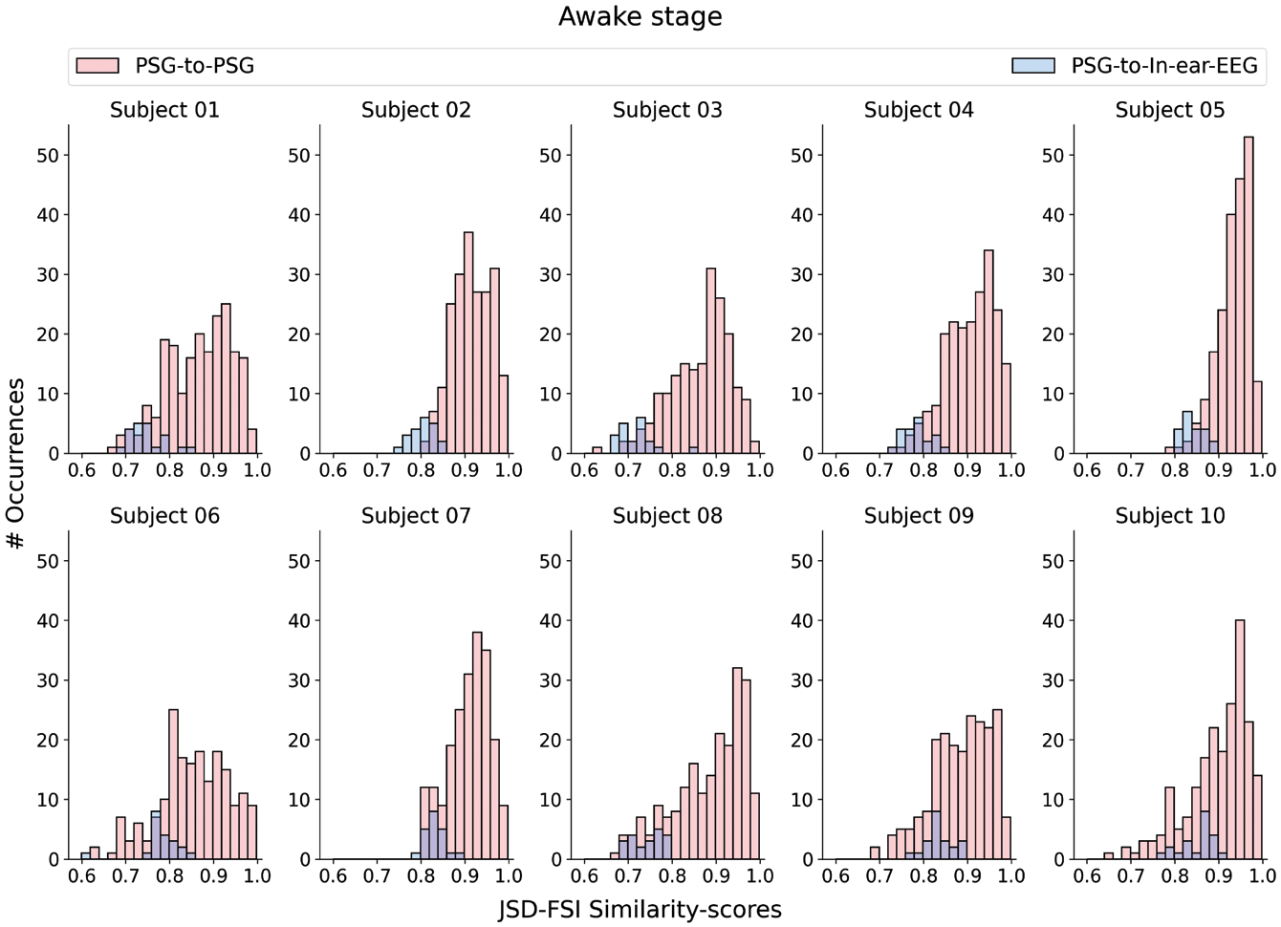
JSD-FSI similarity scores distributions, that is, distributions derived from the PSG-to-in-ear-EEG (histograms in blue) and PSG-to-PSG (histograms in red) comparisons—for each subject in the awake stage.

**Figure 7.**
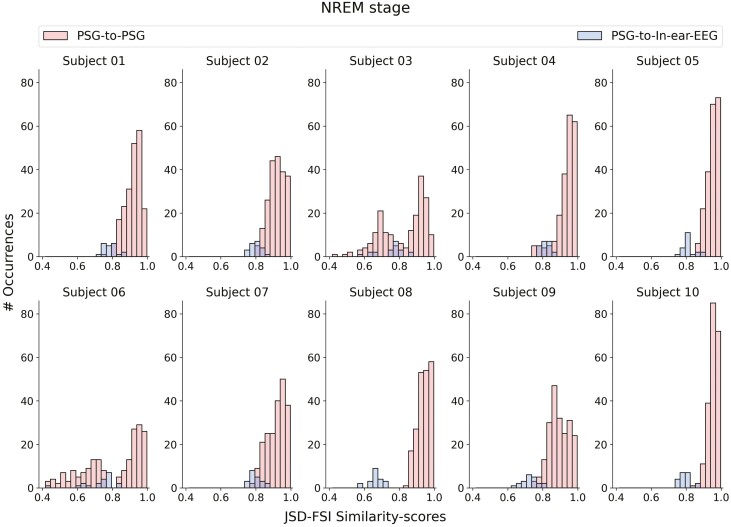
JSD-FSI similarity-scores distributions, that is, distributions derived from the PSG-to-in-ear-EEG (histograms in blue) and PSG-to-PSG (histograms in red) comparisons—for each subject in the NREM sleep stage.

**Figure 8.**
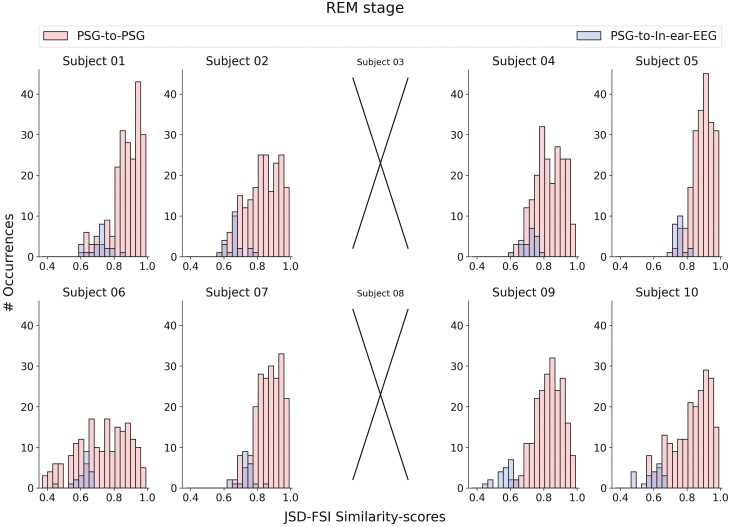
JSD-FSI similarity-scores distributions, that is distributions derived from the PSG-to-in-ear-EEG (histograms in blue) and PSG-to-PSG (histograms in red) comparisons—for each subject in the REM sleep stage. No JSD-FSI similarity scores are reported for subjects 3 and 8.

In Figures 6–8, we show that, for every sleep stage, the blue distributions (i.e. JSD-FSI similarity scores from the PSG-to-In-ear-EEG comparisons) consistently align with the red ones (i.e. JSD-FSI similarity-scores from the PSG-to-PSG comparisons). The only exception occurs for subject 8—no overlap was found between the two distributions in the NREM sleep stage. However, the absence of either a complete or partial overlap between the two distributions (PSG-to-In-ear-EEG and PSG-to-PSG similarity scores) does not directly imply a lack of overlapping information between the two different sources, that is, PSG and in-ear-EEG.

We have to keep in mind that the PSG-to-PSG JSD-FSI similarity scores have been computed mainly to have a reference with which to compare our PSG-to-in-ear-EEG JSD-FSI values. Data from the same source (e.g. PSG signals recorded from the scalp), when compared against each other, should contain close or similar information—resulting in a reference distribution of PSG-to-PSG JSD-FSI similarity scores. Thus, the new in-ear channel, when compared against each of the PSG data sources—that is, channels derived from the scalp—should result in JSD-FSI values close if not equal to our PSG-based reference.

To further analyze and to better interpret the results in Figures 6–8—in Supplementary Material—we report, for each sleep stage, the JSD-FSI similarity score reference distributions computed separately for each PSG data source, that is, scalp-EEG-to-scalp-EEG (Supplementary Figures 7–9) and EOG-to-EOG (Supplementary Figures 10–12).

In Supplementary Figures 7–9, we want to investigate whether the in-ear-EEG shows information similar to the one from the scalp-EEG—by comparing the scalp-EEG-to-In-ear-EEG JSD-FSI distributions with scalp-EEG-to-scalp-EEG reference distributions.

In Supplementary Figures 10–S12 we want to investigate whether the in-ear-EEG shows information similar to the one from the EOG—by comparing the EOG-to-In-ear-EEG JSD-FSI distributions with EOG-to-EOG reference distributions.

As highlighted in the overlapping area in purple, the similarity between the in-ear-EEG and the scalp-EEG channels is higher compared to the one between the in-ear-EEG and the EOG channels—the scalp-EEG-to-In-ear-EEG JSD-FSI distributions overlap with their corresponding reference distributions (scalp-EEG-to-scalp-EEG similarity scores) for almost all subjects and in all the sleep stages.

## Discussion

While evaluating the agreement between the PSG and in-ear-EEG hypnograms scored by the same scorer expert (hypnogram-based comparison analysis), we found a high intrascorer variability. The high variability—or inconsistency between the two different sources—is mainly due to the great uncertainty the scorers had in evaluating the in-ear-EEG signals (see the interscorer variability analysis). The Fleiss’ kappa values between the in-ear-EEG scorers are on average lower—and not consistent—compared to the ones computed on the PSG scorers. We may infer that the in-ear-EEG recordings are harder to score than traditional PSG signals. However, the heightened scoring complexity may not stem from the substandard quality of the in-ear-EEG signal, rather from the innovative nature of the EEG source captured from our ears—distinctly divergent from what scoring experts are used to looking at. The main constraint—compared to the traditional PSG-based scoring procedure—is that the scorers are assigning the sleep stages just relying on a single in-ear-EEG channel. The scorers—hence the physicians—are used to score our sleep considering simultaneously information that comes from different channels.

In our feature-based comparison analysis, we showed a substantial similarity in terms of JSD-FSI score—on average 0.79 ± 0.06 in awake, 0.77 ± 0.07 in NREM, and 0.67 ± 0.10 in REM—between the two different sources. The in-ear-EEG signals are retaining information (in time- and frequency-domain) close to the ones we usually extrapolate through standard PSG derivations. This latter claim is in contrast to what we found following our alternative approach, that is, the hypnogram-based comparison analysis, which mainly relies on the experience and knowledge of the scoring experts. However, considering the agreement metrics between PSG and in-ear EEG consensus, it is evident that the discrepancy between the sleep scoring of these two sources is primarily due to the REM stage.

The high difficulty in scoring in-ear-EEG recordings compared to PSG data may highlight the need for the development of specialized scoring protocols for this new EEG data source, especially for the REM class. While it is true that there is an overlap/similarity in information between in-ear-EEG and PSG—based on sleep feature analysis—it is also possible that this similarity does not necessarily imply that the signal tracings are actually the same.

The robustness of the JSD-FSI similarity scores—and the significance of the similarity values per se—is further validated showing the clear alignment between the PSG-to-In-ear-EEG and PSG-to-PSG JSD-FSI score distributions. The similarity between the in-ear-EEG and any PSG derivation is close to the one we would find between any pair of standard scalp PSG derivations.

The overlap of information between the two signal sources suggests that with further development ear-EEG devices could become a more accessible and less intrusive solution for sleep studies, especially in home settings, compared to traditional PSG setup. The high JSD-FSI scores emphasize the use of mobile ear-EEG solutions as promising alternatives to standard PSG. Our findings are consistent with previous studies exploring the potential of the ear-EEG [2, 8–13]. Indeed, the comparison of the sleep scoring performances between ear-EEG and PSG recordings indirectly represents a measure of similarity between these two sources in the context of sleep analysis.

Some of these studies [9, 12, 13] also highlight the difficulties in scoring the REM stage compared to other classes. Such a difficulty is consistent with our analysis, as we observe significant changes in JSD-FSI score between the sleep stages, with significantly greater values for the awake stage if compared to NREM and REM ones, and for the NREM stage with respect to the REM state. Our results, with the smallest similarity scores for REM sleep, are in line with what we already knew to date: the in-ear-EEG sensors may be not enough in distinguishing REM sleep stage—additional information from EOG signals is needed [3, 10, 16]. This claim is further supported by the results in Supplementary Figures 7–12—where the similarity of the in-ear-EEG with EOG derivations was, overall, lower than the one with scalp-EEG derivations.

Worth highlighting, the primary contribution of this study is a methodological pipeline to quantify the similarity between PSG and in-ear EEG signals. While previous research on in-ear EEG for sleep has focused on developing methodologies for automatic sleep staging, our research aims to first emphasize similarity assessment as a foundational step for validating sleep metrics across any in-ear EEG device.

The main limitation of this preliminary study is that we cannot make any comprehensive consideration regarding JSD-FSI consistency between subjects or channels—i.e. spatial distribution. There is a need to further validate the proposed methodology on a higher number of recordings—eventually involving subjects affected by different sleep disorders—increasing data heterogeneity. A larger and more diverse dataset would also enable a more detailed analysis of sleep stages, including the differentiation of NREM class into N1, N2, and N3 stages. Moreover, future research could explore additional sleep features, such as sleep spindles and K-complexes analyses, as well as connectivity features taking into account interactions between the different brain regions.

## Supplementary Material

zpae087_suppl_Supplementary_Figure_S1

zpae087_suppl_Supplementary_Figure_S2

zpae087_suppl_Supplementary_Figure_S3

zpae087_suppl_Supplementary_Figure_S4

zpae087_suppl_Supplementary_Figure_S5

zpae087_suppl_Supplementary_Figure_S6

zpae087_suppl_Supplementary_Figure_S7

zpae087_suppl_Supplementary_Figure_S8

zpae087_suppl_Supplementary_Figure_S9

zpae087_suppl_Supplementary_Figure_S10

zpae087_suppl_Supplementary_Figure_S11

zpae087_suppl_Supplementary_Figure_S12

zpae087_suppl_Supplementary_Materials

## Data Availability

The dataset from IDUN (BASEC Nr. Req-2022-00105) is not publicly available. The individual identities of participants cannot be inferred from the in-ear-EEG and/or PSG signals. All the data used in this study were collected in accordance with ethical guidelines and with informed consent from the participants. The data are available on request for noncommercial purposes (legal conditions ensuring data privacy and official ethical guidelines compliance will be defined in a “data transfer agreement document,” together with a description of the analysis project).

## Code Availability

To ensure the reproducibility of our analysis, the code has been made openly accessible and is hosted on GitHub.
