# Supplementary figures and images for: Comparison analysis between standard polysomnographic data and in-ear-electroencephalography signals: a preliminary study
Source: Sleep Adv. 2024 Nov 29;5(1):zpae087. doi: 10.1093/sleepadvances/zpae087 (PMC11672114; doi:10.1093/sleepadvances/zpae087)

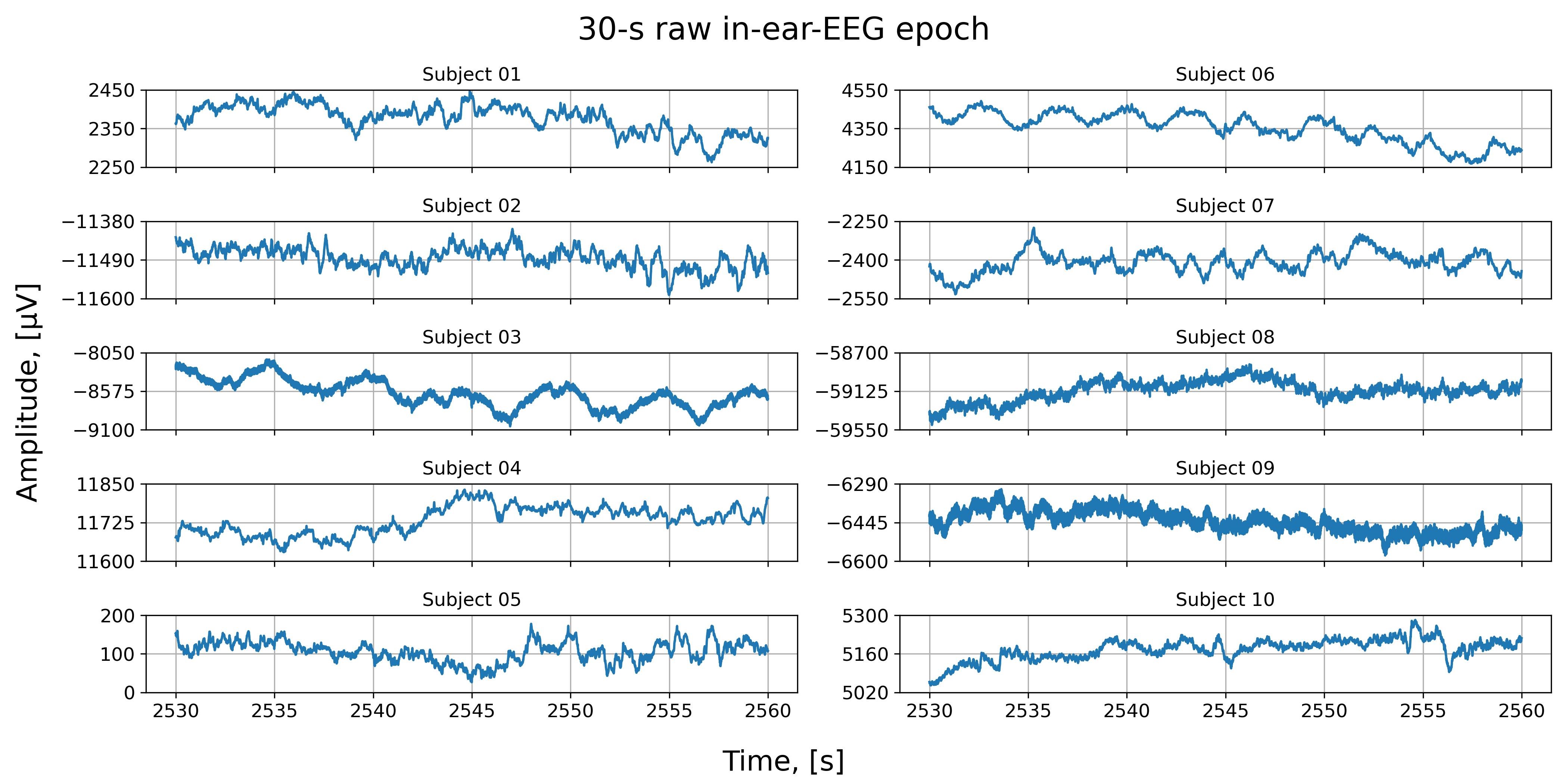

Supplement: zpae087_suppl_Supplementary_Figure_S1 [file zpae087_suppl_supplementary_figure_s1.jpeg]

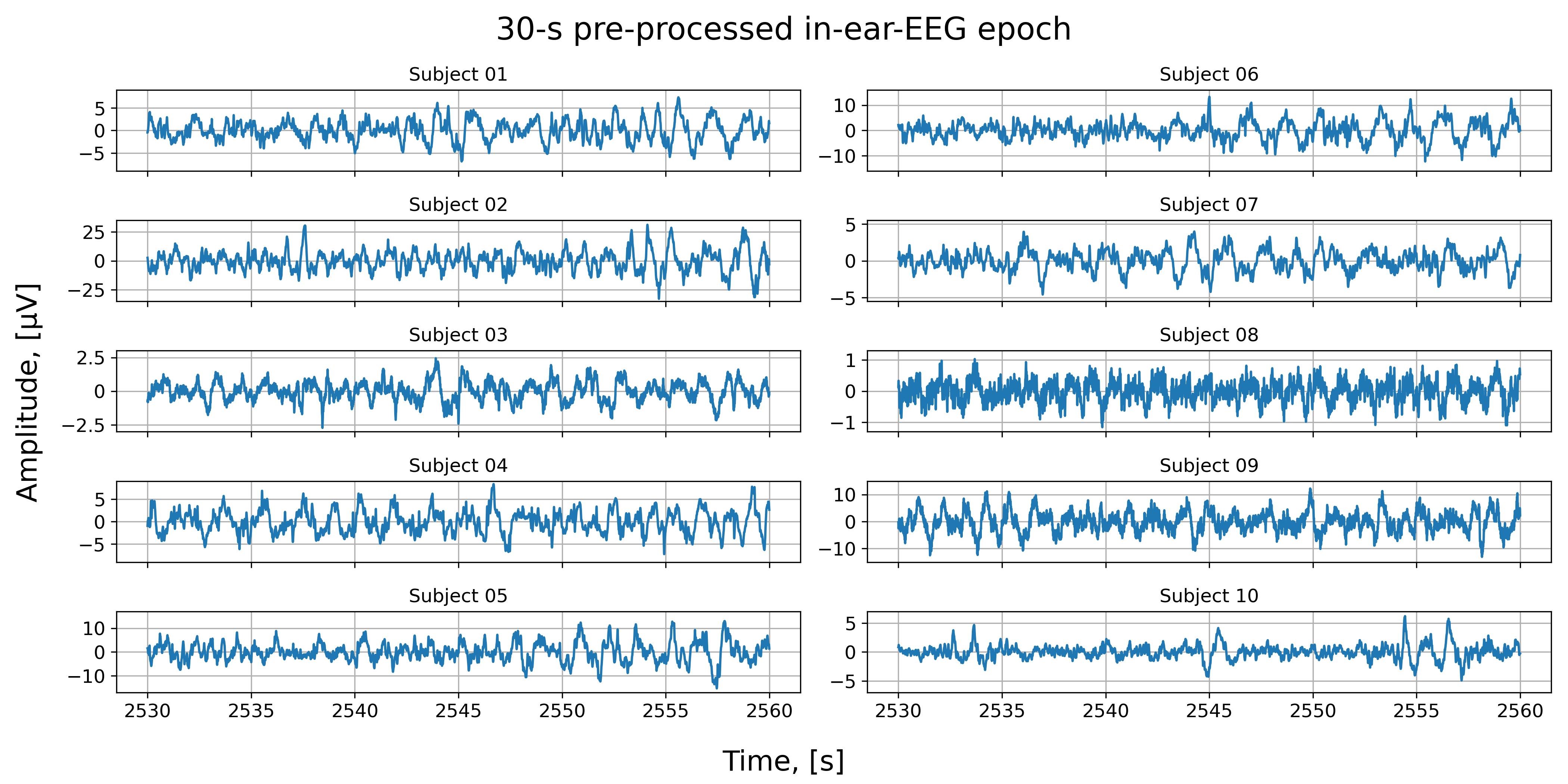

Supplement: zpae087_suppl_Supplementary_Figure_S2 [file zpae087_suppl_supplementary_figure_s2.jpeg]

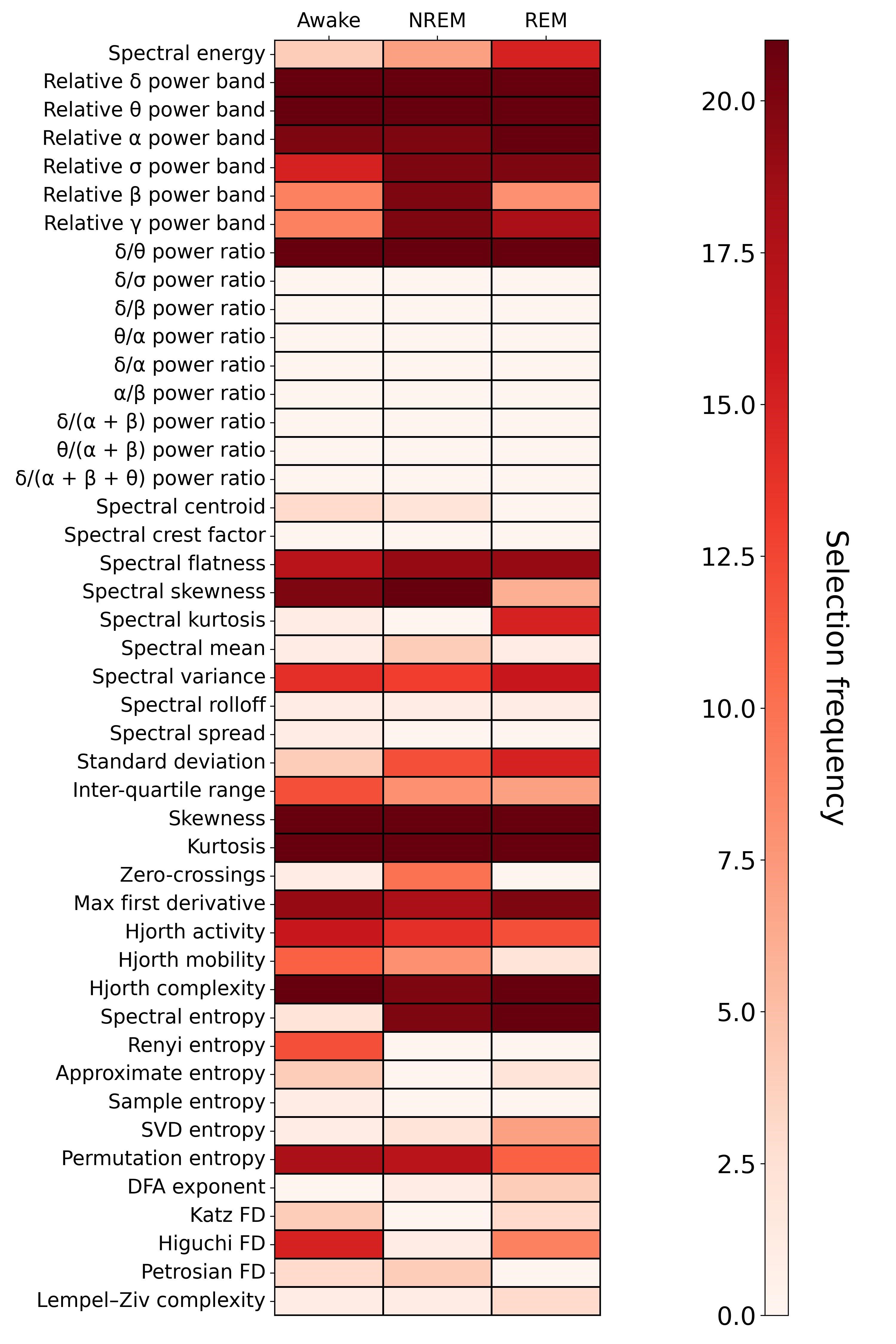

Supplement: zpae087_suppl_Supplementary_Figure_S3 [file zpae087_suppl_supplementary_figure_s3.jpeg]

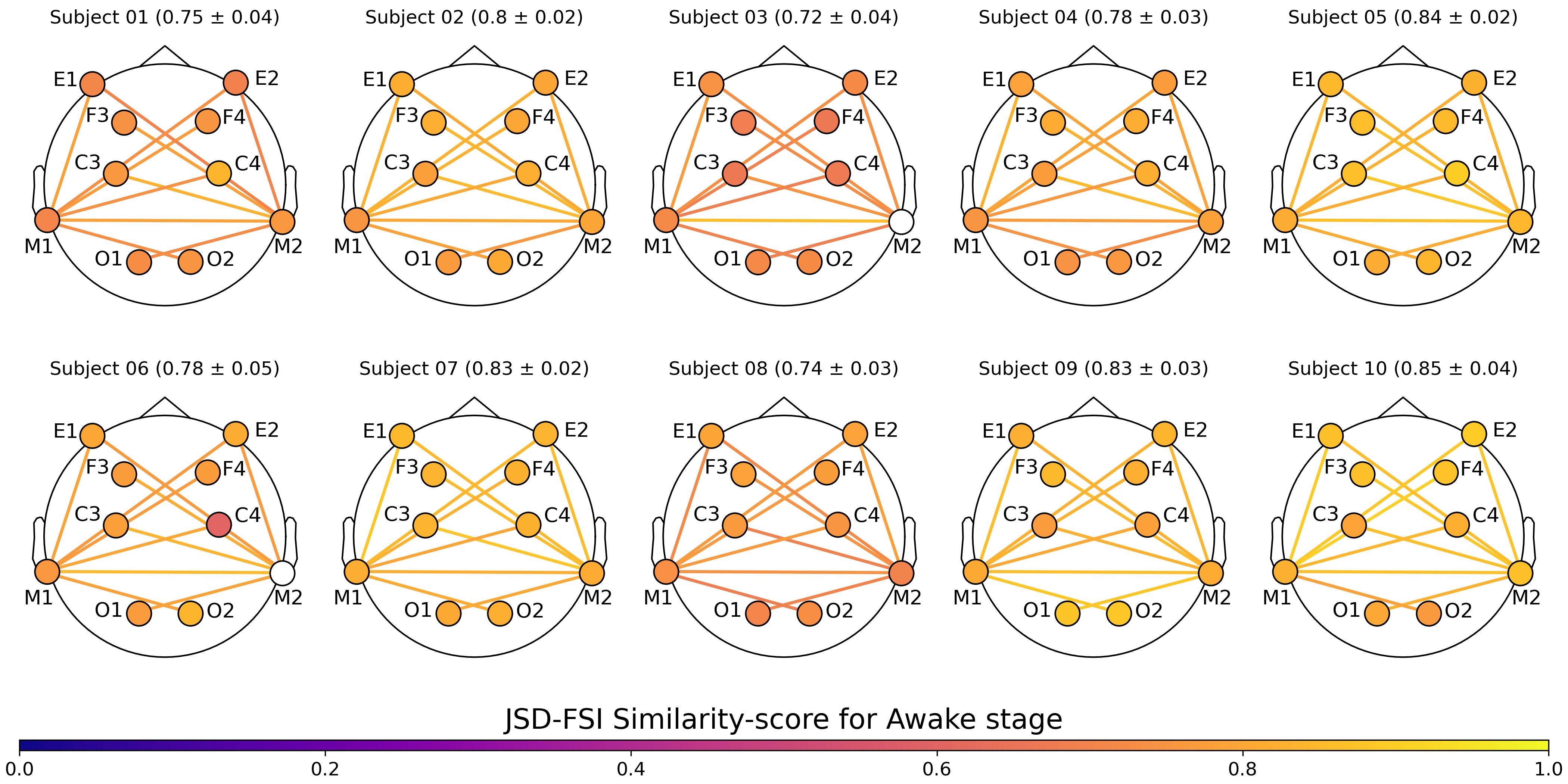

Supplement: zpae087_suppl_Supplementary_Figure_S4 [file zpae087_suppl_supplementary_figure_s4.jpeg]

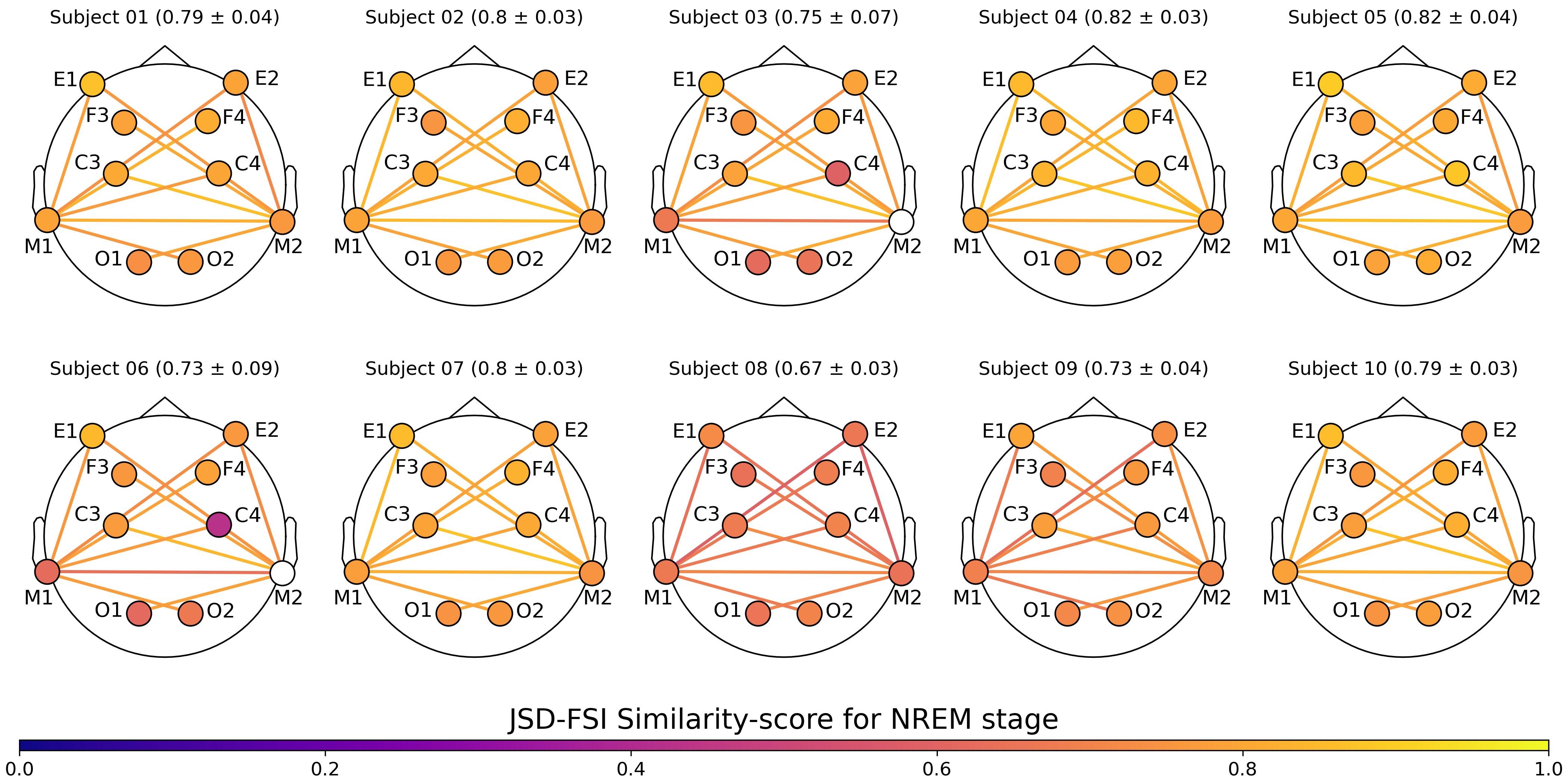

Supplement: zpae087_suppl_Supplementary_Figure_S5 [file zpae087_suppl_supplementary_figure_s5.jpeg]

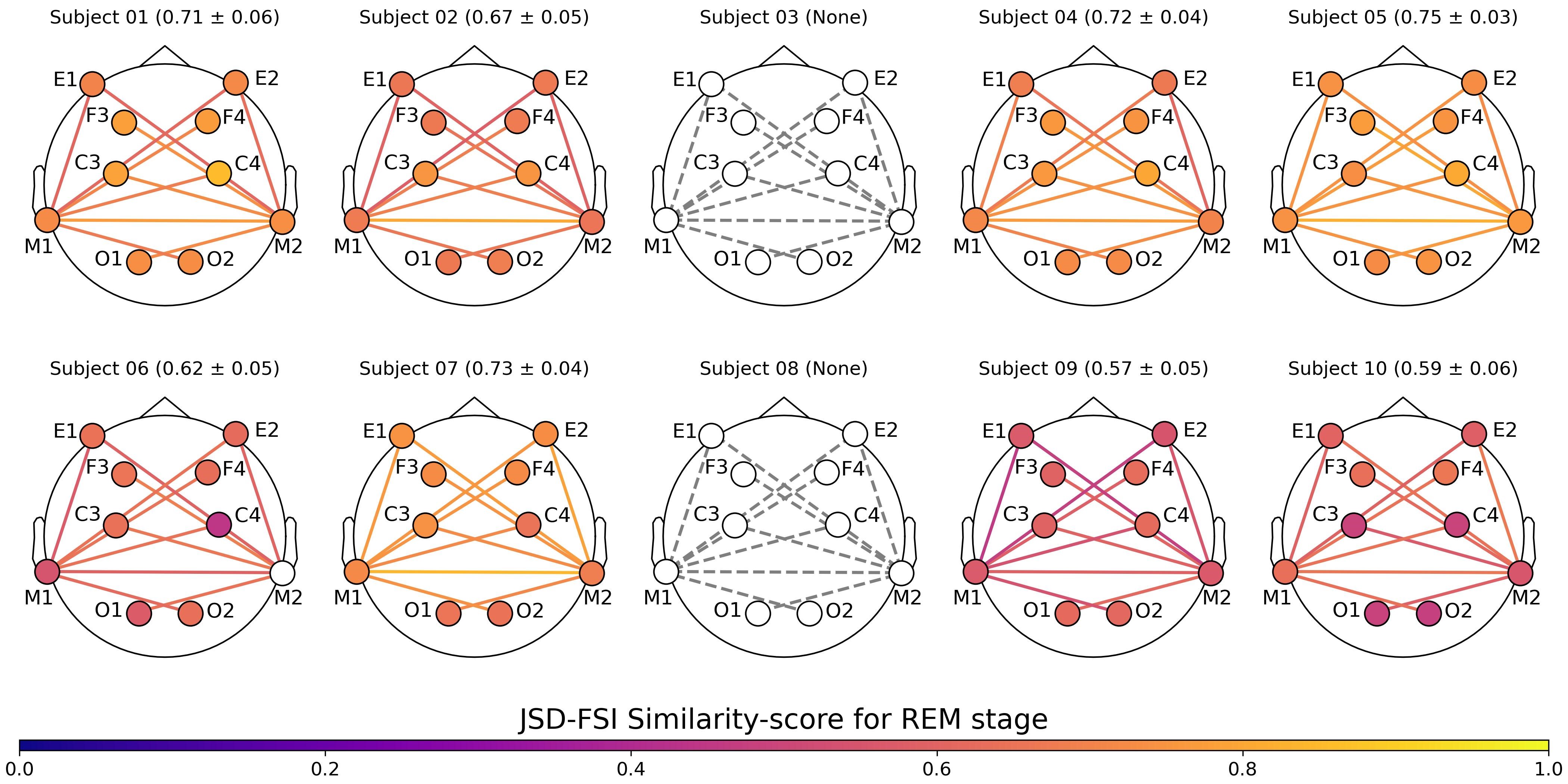

Supplement: zpae087_suppl_Supplementary_Figure_S6 [file zpae087_suppl_supplementary_figure_s6.jpeg]

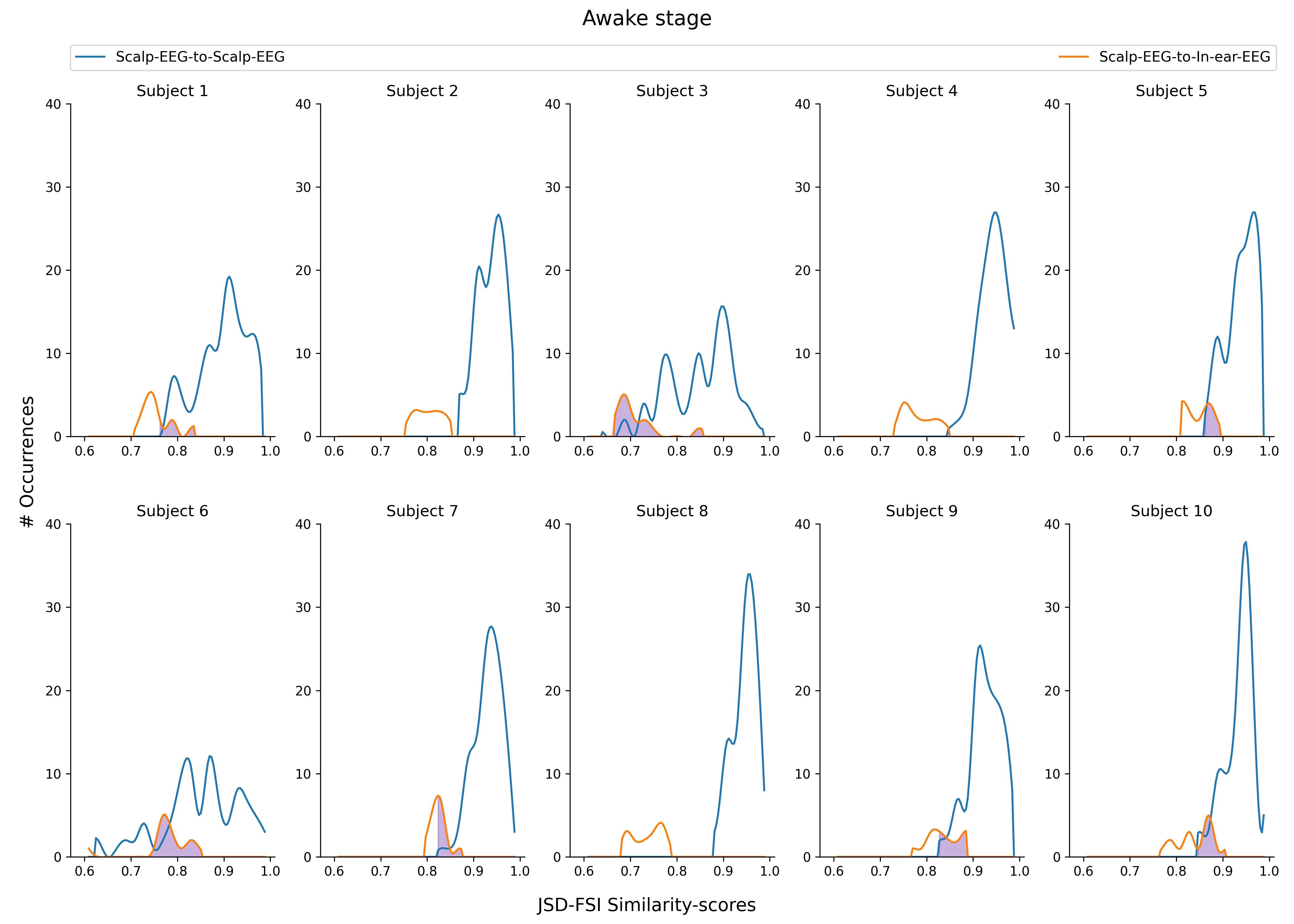

Supplement: zpae087_suppl_Supplementary_Figure_S7 [file zpae087_suppl_supplementary_figure_s7.jpeg]

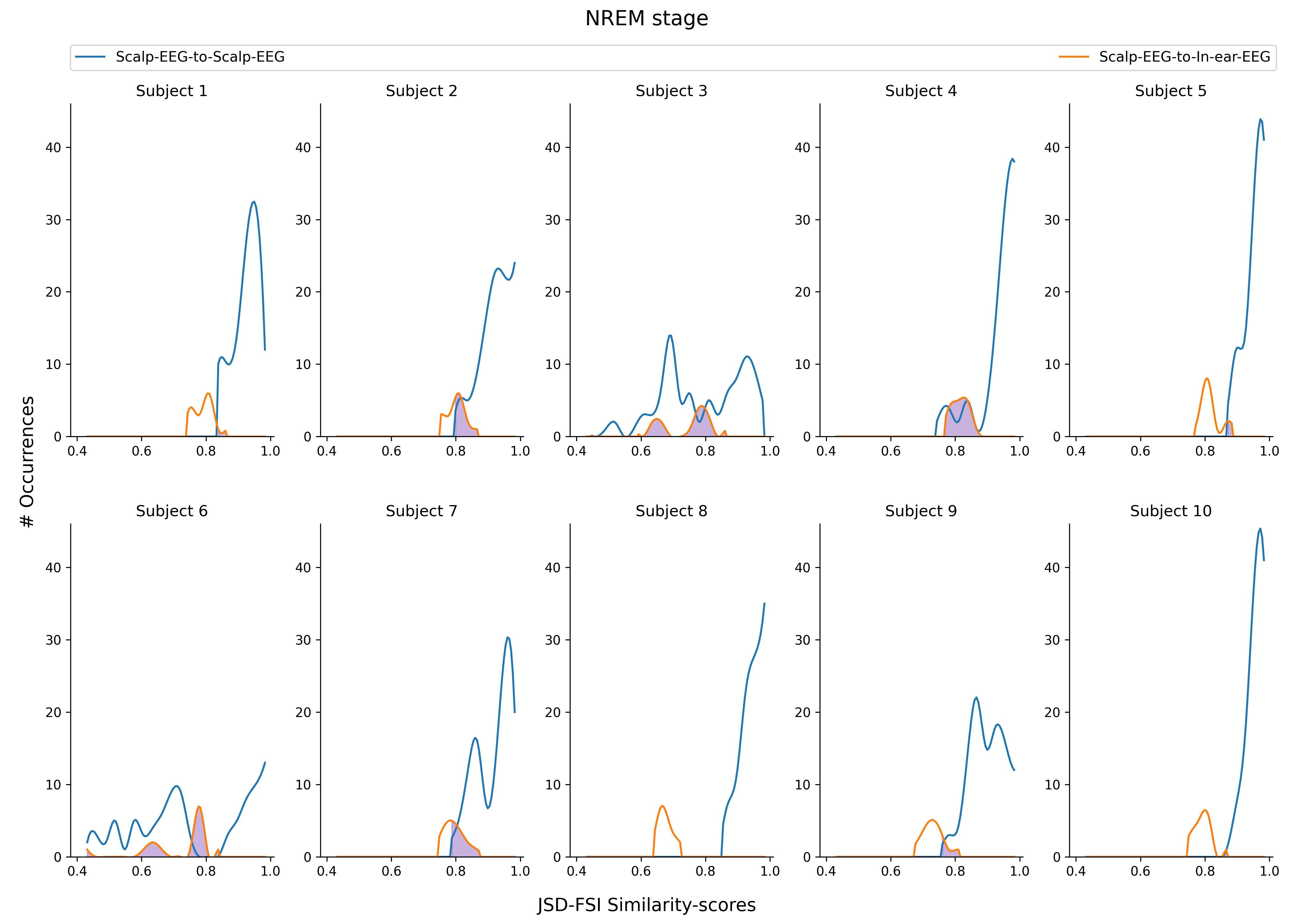

Supplement: zpae087_suppl_Supplementary_Figure_S8 [file zpae087_suppl_supplementary_figure_s8.jpeg]

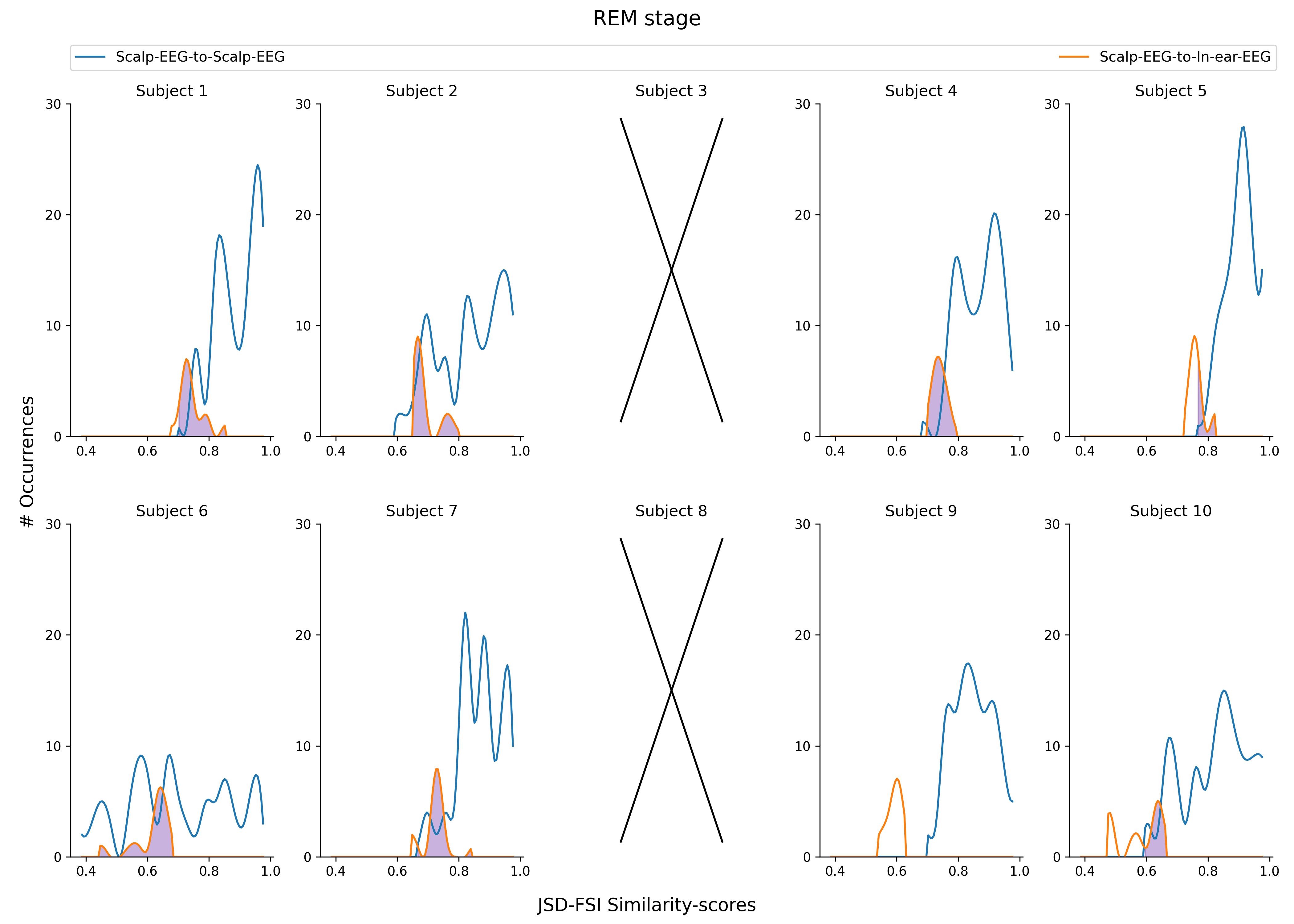

Supplement: zpae087_suppl_Supplementary_Figure_S9 [file zpae087_suppl_supplementary_figure_s9.jpeg]

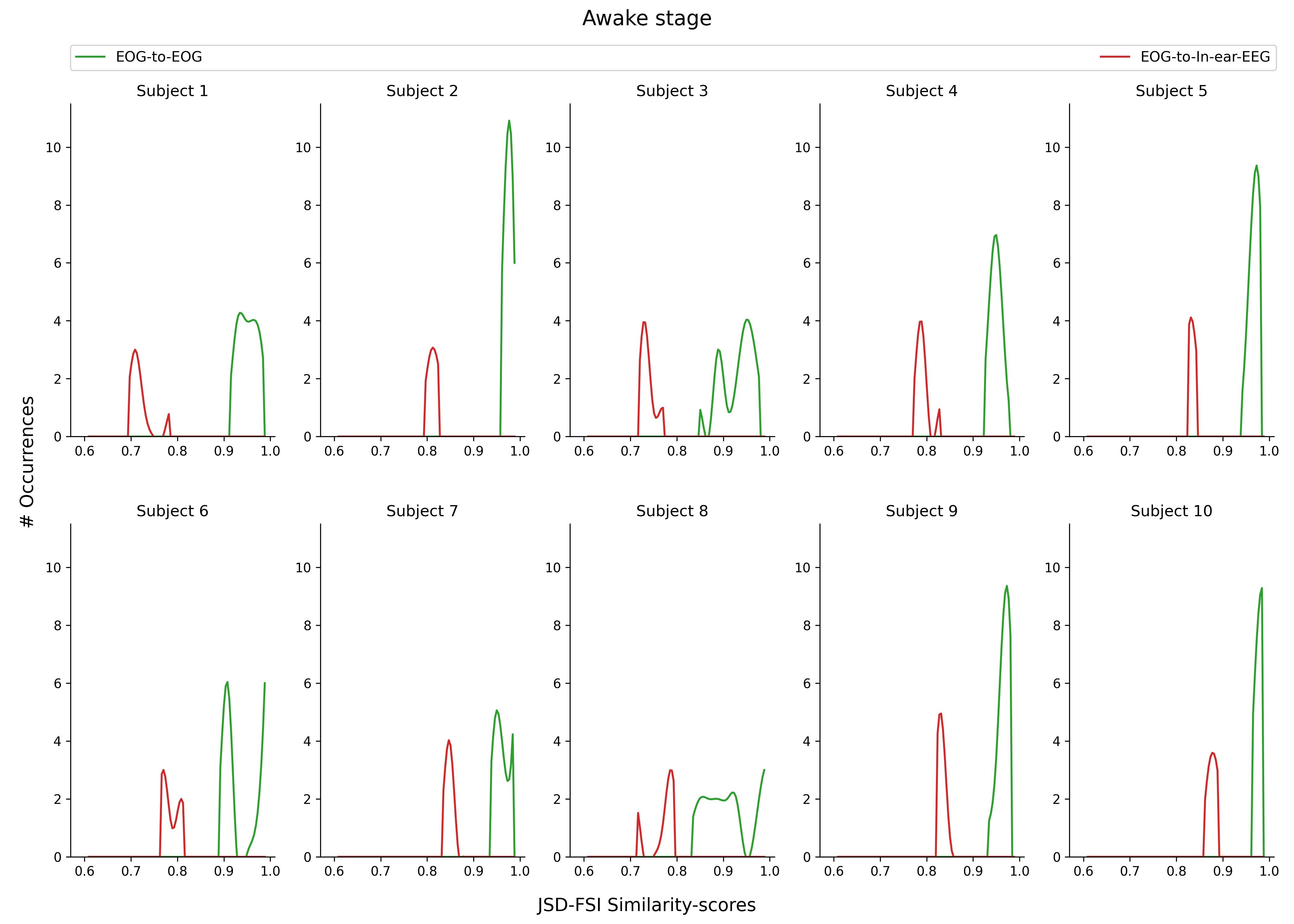

Supplement: zpae087_suppl_Supplementary_Figure_S10 [file zpae087_suppl_supplementary_figure_s10.jpeg]

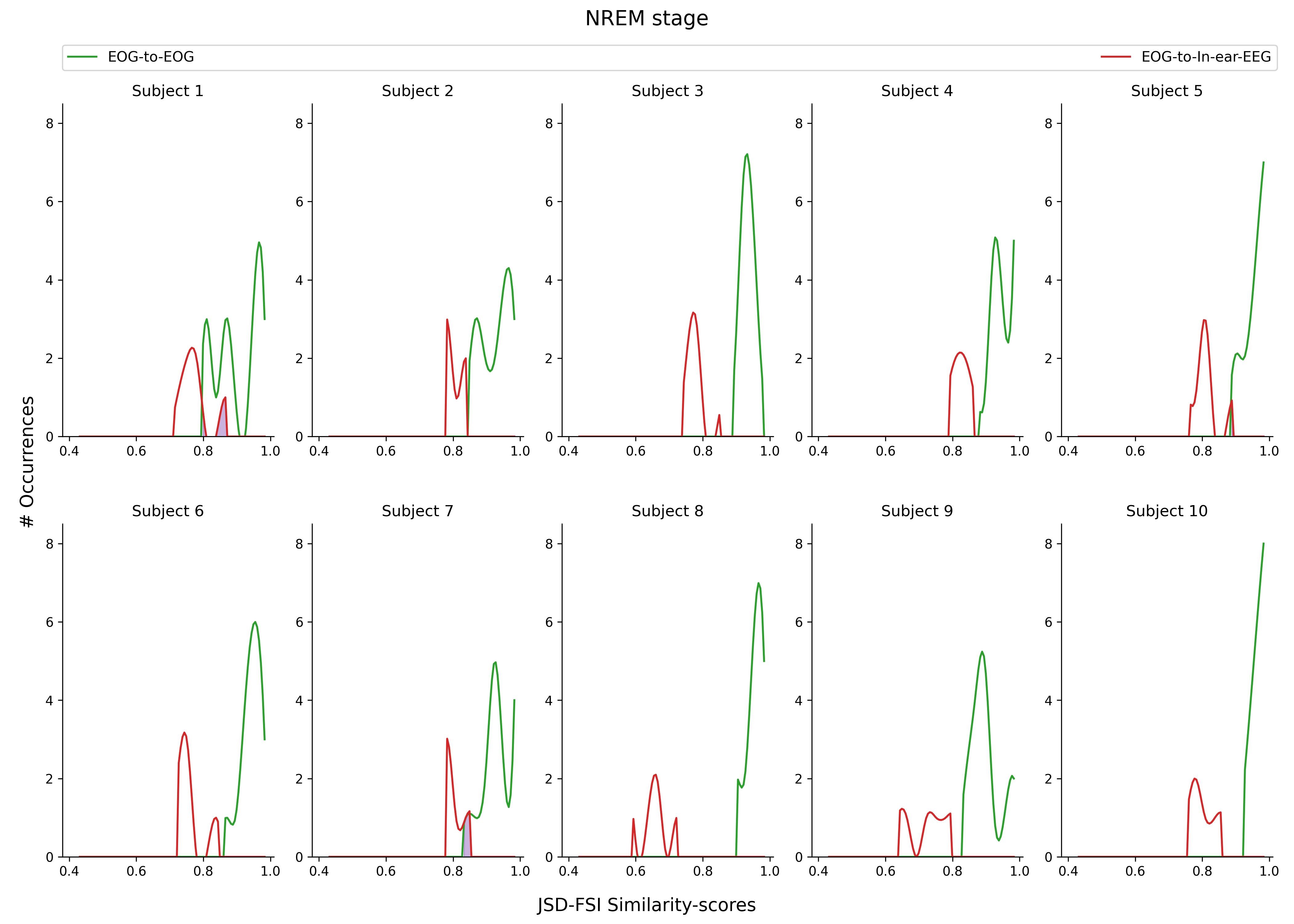

Supplement: zpae087_suppl_Supplementary_Figure_S11 [file zpae087_suppl_supplementary_figure_s11.jpeg]

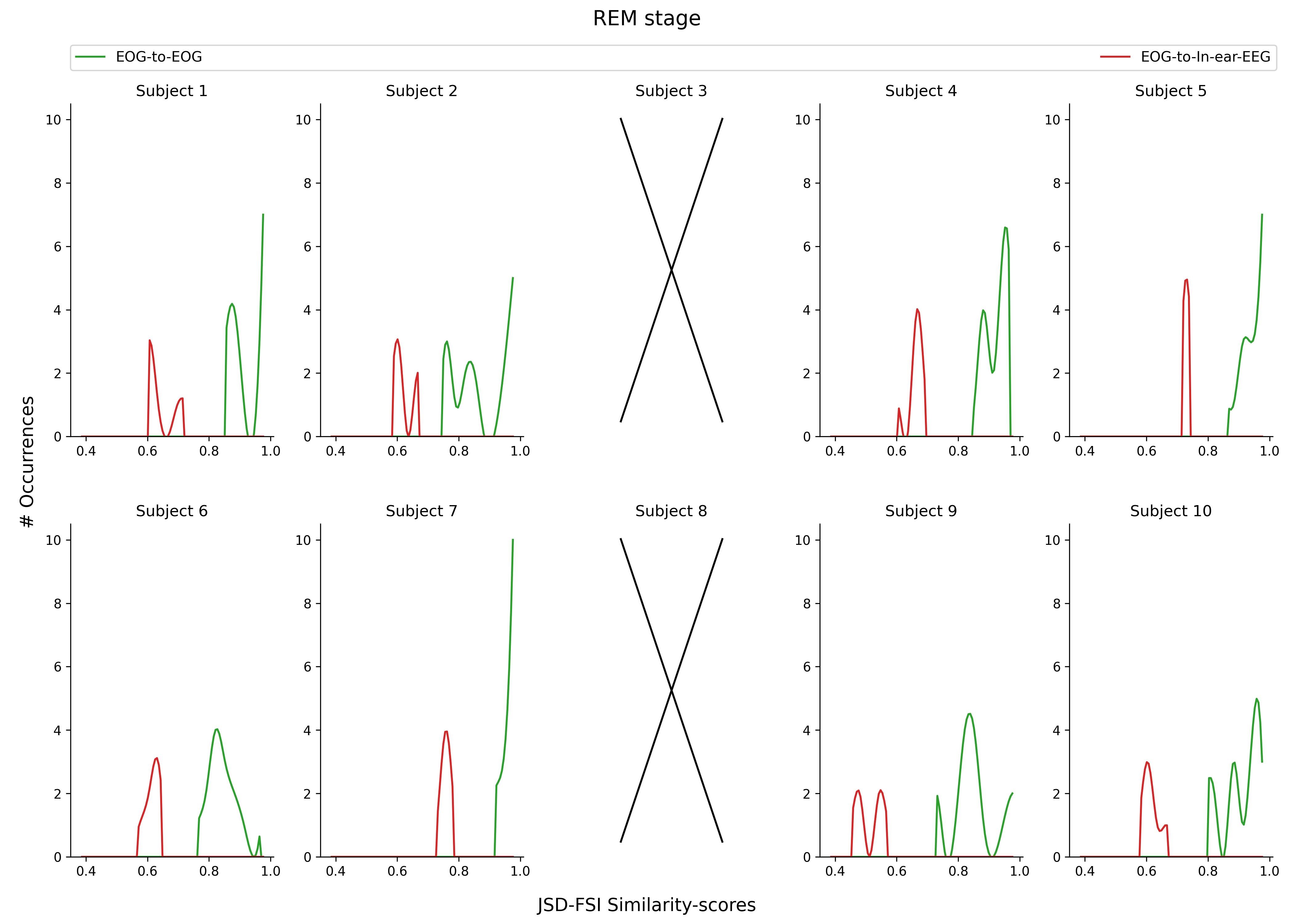

Supplement: zpae087_suppl_Supplementary_Figure_S12 [file zpae087_suppl_supplementary_figure_s12.jpeg]
