# Supplementary material for: Comparison analysis between standard polysomnographic data and in-ear-electroencephalography signals: a preliminary study
Source: Sleep Adv. 2024 Nov 29;5(1):zpae087. doi: 10.1093/sleepadvances/zpae087 (PMC11672114; doi:10.1093/sleepadvances/zpae087)
Supplement: zpae087_suppl_Supplementary_Materials [file zpae087_suppl_supplementary_materials.docx]

# **SUPPLEMENTARY MATERIAL**

# **Comparison analysis between standard polysomnographic data and in-ear-EEG signals: A preliminary study**

## Gianpaolo Palo^1,2,†^, Luigi Fiorillo^1,*, †^, Giuliana Monachino^1,3^, Michal Bechny^1,3^, Michel Wälti^4^, Elias Meier^4^, Francesca Pentimalli Biscaretti di Ruffia^4^, Mark Melnykowycz^4^, Athina Tzovara^3^, Valentina Agostini^2^, and Francesca Dalia Faraci^1^

#### ^1^Institute of Digital Technologies for Personalized Healthcare (MeDiTech), Department of Innovative Technologies, University of Applied Sciences and Arts of Southern Switzerland, Lugano, Switzerland; ^2^Department of Electronics and Telecommunications, Politecnico di Torino, Torino, Italy; ^3^Institute of Computer Science, University of Bern, Bern, Switzerland; ^4^IDUN Technologies AG, Glattpark, Switzerland.

##### Institution where work was performed: Institute of Digital Technologies for Personalized Healthcare (MeDiTech), Department of Innovative Technologies, University of Applied Sciences and Arts of Southern Switzerland, Lugano, Switzerland.

##### †These authors contributed equally to this work. *Corresponding author - Email: [luigi.fiorillo@supsi.ch](mailto:luigi.fiorillo@supsi.ch).

## **SUPPLEMENTARY ANALYSES**

### **Time-domain features**

In the list below we report mathematical details on how to compute each feature.

- ***Standard deviation*** and ***interquartile range*** are statistical measures of dispersion, and are exploited in our study to describe the variation of the electrical activity in the brain [22].
- ***Skewness*** and ***kurtosis*** are the third and fourth central moments in statistics, and are used to characterize the shape of the EEG signals in terms of asymmetry relative to the mean and heaviness of the tails compared to a normal distribution [22].
- The ***maximum first derivative*** and the ***number of zero-crossings*** give information about the depolarization of an EEG recording. The former outlines the highest rate of depolarization in the signal, while the latter estimates its frequency of sign-changes [22].
- ***Approximate entropy*** and ***sample entropy*** are two time series regularity metrics. In general, entropy measures evaluate the complexity of a time series by estimating its intrinsic disorder. The higher the entropy of a signal, the less predictable its evolution.
  The approximate entropy, *ApEn* (1), quantifies the logarithmic likelihood that a signal of length $N$ repeats itself always the same within a certain tolerance $r$. Its estimation includes dividing the signal in sub-segments first of length $d$ and then $\left( d+1 \right)$ before evaluating the correlation integral, $C\left( r \right)$, which expresses how many times each *i-th* sub-segment is akin to all the others [23, 25].

$ApEn\left( d,r,N \right)=\frac{\sum_{i=1}^{N-d+1} log\left[ C_{i}^{d}\left( r \right) \right]}{N-d+1}-\frac{\sum_{i=1}^{N-\left( d+1 \right)+1} log\left[ C_{i}^{d+1}\left( r \right) \right]}{N-\left( d+1 \right)+1}$ *(1)*

The sample entropy, *SpEn* (2), can be taken as a simpler version of the approximate entropy. The difference lies in no longer considering the self-matching for each sub-segment [23].

$SpEn\left( d,r,N \right)=-\frac{1}{N-d+1}log\left[ \frac{\sum_{i=1}^{N-d+1} C_{i}^{d+1}\left( r \right)}{\sum_{i=1}^{N-d+1} C_{i}^{d}\left( r \right)} \right]$ *(2)*

Parameters $d$ and $r$ are respectively set to $2$ and $0.2\cdot SD$ (*SD*: standard deviation of the signal) for both *ApEn* and *SpEn* [24-26].

- ***Singular Value Decomposition entropy (SVDEn)****,* quantifies the complexity of the time series based on the number of eigenvectors needed for its adequate representation. Mathematically, starting from a signal of length $N$, i.e., $\left[ x_{1}, x_{2}, ..., x_{N} \right]$ some delay vectors $y\left( i \right)=\left[ x_{i}, x_{i+\tau}, ..., x_{i+\left( d_{E}-1 \right)\tau} \right]$ are built, having $i=1, 2, ..., N$. Two parameters are involved, i.e., the embedded dimension ($d_{E}$) and the delay ($\tau$), which are respectively set to $3$ and $1$, as recommended for short duration EEG signals [26, 27]. SVD factorization is then performed on the embedded matrix $Y=\left[ y\left( 1 \right), y\left( 2 \right), ..., y\left( N-\left( d_{E}-1 \right)\tau\right) \right]$, resulting in $M$ singular values $\left( \sigma_{1}, \sigma_{2}, ..., \sigma_{M} \right)$. Hence, SVD entropy is computed on normalized singular values, such that $\underline{\sigma_{i}}=\sigma_{i}/\sum_{j=1}^{M} \sigma_{j}$, following (3) [27].

$SVDEn=-\sum_{i=1}^{M} \underline{\sigma_{i}}{log}_{2}\left( \underline{\sigma_{i}} \right)$ *(3)*

- ***Permutation entropy (PermEn)***, involves the partition of the time series of length $N$ into a matrix of overlapping column vectors of length $D$. The overlap is defined by a delay parameter ($\tau$). In this work, $D=3$ and $\tau=1$ [26, 28]. Column vectors are then mapped into permutations ($\pi$) capturing the ordinal rankings of data. Hence, permutation entropy is calculated as shown in (4), where $p_{i}$ is the relative frequency of the *i-th* permutation ($\pi_{i}$) and is evaluated as the number of times $\pi_{i}$ is found in the signal over the total number of sequences [26, 28].

$PermEn=-\sum_{i=1}^{D!} p_{i}{log}_{2}\left( p_{i} \right)$ *(4)*

- ***Lempel-Ziv complexity (C)****,* evaluates the randomness of the time series. First, the signal of length $N$ is transformed into a binary sequence using coarse-graining. This is performed by assigning 0 and 1 to values respectively below and above a threshold. The latter is defined as the median of the window time series analyzed for its robustness to possible outliers [29]. While inspecting the signal from left to right, the overall number of distinct subsequences, $c\left( N \right)$, of consecutive characters is assessed. To get a measure that is independent of the sequence length, Lempel-Ziv complexity is defined as the normalized version of $c\left( N \right)$ (5).

$C\left( N \right)=c\left( N \right)\frac{{log}_{2}\left( N \right)}{N}$ *(5)*

- ***Detrended Fluctuation Analysis* (*DFA*)** **exponent**, $\alpha$, measures the degree of long-term statistical dependencies or intrinsic self-similarity in time series. This feature is used to quantify the long-range correlation properties of the EEG signal by evaluating possible self-similar patterns in the electrical activity of the brain. First, the time series $x$ of length $N$ is integrated to define the ‘*accumulated walk*’, $y\left( k \right)$ (6).

$y\left( k \right)=\sum_{i=1}^{k} \left[ x\left( i \right)-\underline{x} \right]$  *(6)*

where $x\left( i \right)$ is the sequence at the *i-th* sample, and $\underline{x}$ is the mean of the entire time series. Hence, $y\left( k \right)$ is divided into sub-sequences of equal length $n$, and a least-squares line fitting the data within each sub-sequence is evaluated. The y-coordinate of the latter, $y_{n}\left( i \right)$, describes the local trend of each sub-sequence. The Root-Mean-Square (RMS) fluctuation, $F\left( n \right)$ (7), is then calculated by integrating and detrending $y\left( k \right)$.

$F\left( n \right)=\sqrt{\frac{1}{N}\sum_{i=1}^{N} \left[ y\left( i \right)-y_{n}\left( i \right) \right]^{2}}$  *(7)*

The whole process is iterated over all the possible lengths, $n$. The DFA exponent is defined as the slope of the best-fitting line to the distribution of the RMS fluctuation as a function of the segment size on a log-log scale.
Whether $\alpha>0.5$, the time series is characterized by long-range correlations, while $\alpha<0.5$ outlines that the signal is *anti-persistent, i.e.,* it shows a negative correlation. The case $\alpha=0.5$ indicates that changes in time series are random and uncorrelated with each other, thus the signal can be modeled as *white noise* [30, 31].

- ***Hjorth parameters***, i.e., ***activity*** $\boldsymbol{H}_{\boldsymbol{A}}$**, *mobility*** $\boldsymbol{H}_{\boldsymbol{M}}$**, and *complexity*** $\boldsymbol{H}_{\boldsymbol{C}}$, are statistical functions based on the first and second derivatives of time series useful to characterize the dynamics of the brain function. Let $x$ be an EEG signal of length $N$ and $\underline{x}$ be its mean value, $H_{A}$ (8) provides information about its energy; $H_{M}$ (8) is introduced to assess the variability of its frequency over time; and $H_{C}$ (8) gives insights into the complexity of its waveform in terms of amplitude and frequency with respect to a common sine wave [32].

$H_{A}=var\left[ x\left( t \right) \right]=\frac{\sum_{i=1}^{N} \left( x_{i}-\underline{x} \right)^{2}}{N}$; $H_{M}=\sqrt{\frac{var\left[ \frac{d}{dt}x\left( t \right) \right]}{H_{A}}}$; $H_{C}=\frac{H_{M}\left[ \frac{d}{dt}x\left( t \right) \right]}{H_{M}\left[ x\left( t \right) \right]}$  *(8)*

- ***Katz***, ***Higuchi***, and ***Petrosian*** fractal dimensions estimate the fractality of the time series by measuring its complexity and self-similarity over different scales of observation [35].
  According to Katz fractal dimension, ${FD}_{K}$, the complexity is defined based on the deviation of the trajectory of the time series from the simplest path, i.e., the straight line between the first and last points (9).

${FD}_{K}=\frac{log\left( n \right)}{log\left( n \right) + log\left( \frac{d}{L} \right)}$ *(9)*

where $d$ is the farthest distance among all those measured between the first point of the signal and all the others; $L$ is the total length of the waveform and is calculated as the sum of the distances between successive points of the signal; and $n=L/a$ is the number of units that compose the time series. In particular, to avoid different units leading to different fractal dimensions, a general unit, $a$, is defined as the average distance between subsequent points [35, 36].

Using Higuchi fractal dimension, ${FD}_{H}$*,* the complexity is assessed by analyzing how the pattern of the signal changes when sampled at different time intervals. First, starting from the time series $x=x\left( 1 \right), x\left( 2 \right), ..., x\left( N \right)$ of length $N$, $k$ new sequences are defined following (10), where $m=1, 2, ..., k$ is the initial time value; $k\in\left[ 1, k_{max} \right]$ is the discrete time delay; and $\lfloor a\rfloor$ indicates the integer part of $a$*.* In this study, $k=10$ [33, 36].

$x_{k}^{m}=\left[ x\left( m \right), x\left( m+k \right), x\left( m+2k \right), ..., x\left( m+\lfloor\frac{N-m}{k}\rfloor k \right) \right]$ *(10)*

The length of each $x_{k}^{m}$ is evaluated, $L_{m}\left( k \right)$ (11), before averaging all those associated with the same delay, $k$ (12). Therefore, ${FD}_{H}$ is determined as the slope of the least squares linear best fit to the distribution of $L\left( k \right)$ versus $1/k$ on a double logarithmic scale.

$L_{m}\left( k \right)=\frac{N-1}{k} \left[ \frac{1}{\lfloor\frac{N-m}{k}\rfloor}\sum_{i=1}^{\lfloor\left( N-m \right)/k\rfloor} \left| x\left( m+ik \right)-x\left( m+\left( i-1 \right)k \right) \right| \right] \frac{1}{k}$ *(11)*

$L\left( k \right)=\sum_{m=1}^{k} L_{m}\left( k \right) \propto k^{-{FD}_{H}}$ *(12)*

Similarly to ${FD}_{K}$*,* Petrosian fractal dimension, ${FD}_{P}$, quantifies the complexity of the time series in relation to how much it deviates from a straight line. In particular, given a signal of length $N$, Petrosian’s definition (13) focuses on the number of sign-changes in its first derivative, thus emphasizing the rate of slope reversals [34, 35].

${FD}_{P}=\frac{log\left( N \right)}{log\left( N \right) + log\left( \frac{N}{N + 0.4N_{\Delta}} \right)}$ *(13)*

###

### **Frequency-domain features**

In the list below we report mathematical details on how to compute each feature.

- ***Spectral energy*** represents the total energy of the time series and is defined by integrating the Power Spectral Density (PSD) of the signal.
  ***Relative powers*** of all the EEG frequency bands, i.e., ***delta*** ($\delta, 0.5-4 Hz$), ***theta*** ($\theta, 4-8 Hz$), ***alpha*** ($\alpha, 8-12 Hz$), ***sigma*** ($\sigma, 12-16 Hz$), ***beta*** ($\beta, 16-30 Hz$), and ***gamma*** ($\gamma, 30-35 Hz$) are also calculated. These estimate how the signal energy is distributed across its several frequency components.
  In addition, several ***ratios between frequency bands*** are included as features, i.e., $\delta/\theta$, $\delta/\sigma$, $\delta/\beta$, $\theta/\alpha$, $\delta/\alpha$, $\alpha/\beta$, $\delta/(\alpha+\beta)$, $\theta/(\alpha+\beta)$, $\delta/(\alpha+\beta+\theta)$.
- Unlike previous entropy metrics, ***spectral entropy****,* ***SpecEn*** (14)*,* and ***Renyi entropy****,* ***RenyiEn*** (15) are measured in the frequency domain. In particular, they assess the complexity of the EEG signal by working on its normalized power spectrum [40].

$SpecEn = -\sum_{f} p\left( f \right)log\left[ p\left( f \right) \right]$ *(14)*

$RenyiEn = -log\left[ \sum_{f} p^{2}\left( f \right) \right]$ *(15)*

- ***Spectral centroid*** represents the center of mass of the spectrum of the time series and is calculated as the frequency-weighted mean of the PSD of the signal [41, 42].
- ***Spectral flatness*** is computed as the ratio of the geometric mean of the power spectrum to its arithmetic mean [41]. It evaluates how much noise-like the signal is [42].
- ***Spectral spread*** quantifies the dispersion of the power spectrum around its spectral centroid. Numerically, it is given by the weighted mean of the PSD of the signal and the weights are defined as the squared differences between the spectral centroid and all the examined frequencies [42].
- ***Spectral crest factor*** provides information about how extreme the peaks of the spectrum of the signal are. It is evaluated as the ratio of the maximum power spectrum to the mean PSD of the signal [43].
- ***Spectral roll-off*** is defined as the frequency beneath which a certain percentage of the overall energy lies. In this study, this feature is determined relative to 85% of the energy [44].
- The four ***spectral central moments*** in statistics, i.e., ***mean***, ***variance***, ***skewness***, and ***kurtosis***, provide insights into the shape and distribution of the PSD of the signal.

### **Feature selection**

The feature selection algorithm we choose relies on pairwise feature similarity, which is evaluated using the maximal information compression index (MICI). This metric has been shown to outperform two other commonly used feature similarity measures [45, 47].

Let $\Sigma$ be the two-by-two covariance matrix of features x and y, $\lambda_{2}$ is defined as the smallest eigenvalue of $\Sigma$ (16, 17).

$\lambda_{2}\left( x,y \right)=\frac{1}{2}\left[ var\left( x \right)+var\left( y \right)-\sqrt{A\left( x,y \right)} \right]$ $\left( 16 \right)$

$A\left( x,y \right)=\left[ var\left( x \right)+var\left( y \right) \right]^{2}-4var\left( x \right)var\left( y \right)\left[ 1-{\rho\left( x,y \right)}^{2} \right]$ $\left( 17 \right)$

where $var$ stands for variance and $\rho$ represents Pearson’s correlation coefficient.

In particular, what is used here is a modified version of the MICI, as it gets normalized by the sum of the variances of the features to not have sensitivity to the features’ scale (18) [47].

$\lambda_{2, norm}\left( x,y \right)=\frac{\lambda_{2}\left( x,y \right)}{var\left( x \right) + var\left( y \right)}$ $\left( 18 \right)$

The Feature Selection using Feature Similarity (FSFS) algorithm [45] is based on the k-nearest neighbors (kNN) principle, i.e., it divides the initial feature subset into homogeneous clusters, before selecting only the most representative feature from each such cluster. Iteratively, the algorithm selects only the feature showing the most compact subset, i.e., the lowest distance from its farthest neighbor, thus removing all the k-nearest ones.

A constant error threshold, $\varepsilon$, is defined by the MICI value between the feature selected at the first iteration and its k-th neighbor. This is used to adapt the algorithm in such a way that at each iteration whenever the smallest $\lambda_{2}$ is lower than $\varepsilon$, the number of k-nearest neighbors (k) decreases [45].

The best initial value for k is set according to two metrics, i.e., the representation entropy ($H_{R}$) (19) and the redundancy rate ($RR$) (20). The former quantifies the information compression. Higher values are linked to a more balanced feature selection process, i.e., lower redundancy within the feature subset [45, 46].

$H_{R}=-\sum_{i=1}^{N} \hat{\lambda}_{i}log\hat{\lambda}_{i}$ $\left( 19 \right)$

where $\hat{\lambda}_{i}$ is the normalized eigenvalue of the covariance matrix of the feature subset of size N, having i=1, 2, …, N and $\hat{\lambda}_{i}=\lambda_{i}/\sum_{i=1}^{N} \lambda_{i}$.

The redundancy rate evaluates the redundant information within the feature subset with larger values indicating a strong correlation among features [46, 48].

$RR=\frac{1}{N\left( N-1 \right)}\sum_{f_{i},f_{j}\in F, i>j} \rho_{i,j}$ $\left( 20 \right)$

where $\rho$ is the Pearson’s correlation coefficient measured for each pair of features ($f$) of the target subset ($F$) of size N. The best number of k-nearest neighbors is independently found for each sleep stage and for each pair of PSG and in-ear-EEG channels as the one that maximizes $H_{R}$. Hence, the chosen k-value is validated using the RR metric. First, we identify the k-value related to the maximum of the representation entropy, $H_{R, max}$ - once verified that the latter is greater than the reference value, i.e., the representation entropy evaluated on the initial feature subset, $H_{R, ref}$. Hence, we validate the chosen k-value, $k_{0}$, by verifying that the corresponding redundancy rate, $RR\left( k_{0} \right)$, is lower than the reference value, i.e., the redundancy rate evaluated on the initial feature subset, ${RR}_{ref}$. If either of these two conditions $- H_{R}\left( k_{0} \right) = H_{R, max} > H_{R, ref}$ and $RR\left( k_{0} \right) < {RR}_{ref} -$ is not met, the analysis is iterated considering the next highest $H_{R}$ value; and if both conditions are never simultaneously fulfilled, the initial number of k-nearest neighbors is set equal to zero.

## **SUPPLEMENTARY TABLES**

**Table S1.**

$Soft$*-*$Agreement$ values computed on each of the three scorers and for each subject on the PSG data source. For each subject, we report the most reliable scorer with the corresponding cell with edges highlighted in bold.

| Soft-agreement for PSG scorers | | | |
| --- | --- | --- | --- |
|  | Scorer 1 | Scorer 2 | Scorer 3 |
| Subject 1 | 0.9366 | 0.9673 | 0.9836 |
| Subject 2 | 0.8840 | 0.9672 | 0.9540 |
| Subject 3 | 0.9443 | 0.9550 | 0.8737 |
| Subject 4 | 0.9588 | 0.9897 | 0.9670 |
| Subject 5 | 0.8261 | 0.9855 | 0.9524 |
| Subject 6 | 0.8950 | 0.9391 | 0.8466 |
| Subject 7 | 0.9582 | 0.9940 | 0.9681 |
| Subject 8 | 0.8929 | 0.9841 | 0.9167 |
| Subject 9 | 0.9791 | 0.9248 | 0.9687 |
| Subject 10 | 0.9561 | 0.9728 | 0.9477 |
| **Averaged** | **0.9231** $\pm$ **0.0447** | **0.9680** $\pm$ **0.0215** | **0.9379** $\pm$ **0.0427** |

**Table S2.**

$Soft$*-*$Agreement$ values computed on each of three scorers and for each subject on the in-era-EEG data source. For each subject, we report the most reliable scorer with the corresponding cell with edges highlighted in bold.

| Soft-agreement for in-ear-EEG scorers | | | |
| --- | --- | --- | --- |
|  | Scorer 1 | Scorer 2 | Scorer 3 |
| Subject 1 | 0.8773 | 0.9121 | 0.9550 |
| Subject 2 | 0.8709 | 0.8840 | 0.9540 |
| Subject 3 | 0.8522 | 0.8244 | 0.9465 |
| Subject 4 | 0.8784 | 0.8247 | 0.9443 |
| Subject 5 | 0.8468 | 0.9006 | 0.9379 |
| Subject 6 | 0.7479 | 0.8382 | 0.9454 |
| Subject 7 | 0.9821 | 0.8825 | 0.9203 |
| Subject 8 | 0.8651 | 0.7897 | 0.8452 |
| Subject 9 | 0.8225 | 0.9353 | 0.9165 |
| Subject 10 | 0.7762 | 0.9519 | 0.8891 |
| **Averaged** | **0.8519** $\pm$ **0.0602** | **0.8743** $\pm$ **0.0504** | **0.9254** $\pm$ **0.033** |

**Table S3.**

List of all the extracted time-domain features. Abbreviations: Detrended Fluctuation Analysis (DFA); Singular Value Decomposition (SVD). ***** features depending on amplitude, thus computed on normalized signals.

| **Time-domain features** | | |
| --- | --- | --- |
| Standard deviation* | DFA exponent | Hjorth activity* |
| Skewness | Approximate entropy | Hjorth mobility |
| Kurtosis | Sample entropy | Hjorth complexity |
| Maximum first derivative* | SVD entropy | Katz fractal dimension |
| Interquartile range* | Permutation entropy | Higuchi fractal dimension |
| Number of zero-crossings | Lempel-Ziv complexity | Petrosian fractal dimension |

**Table S4.**

List of all the extracted frequency-domain features. ***** features depending on amplitude, thus computed on normalized signals.

| **Frequency-domain features** | | |
| --- | --- | --- |
| Spectral energy* | $\delta/\theta$ power ratio | Spectral centroid |
| Relative $\delta$ power band | $\delta/\sigma$ power ratio | Spectral crest factor |
| Relative $\theta$ power band | $\delta/\beta$ power ratio | Spectral flatness |
| Relative $\alpha$ power band | $\delta/\alpha$ power ratio | Spectral roll-off |
| Relative $\sigma$ power band | $\theta/\alpha$ power ratio | Spectral spread |
| Relative $\beta$ power band | $\alpha/\beta$ power ratio | Spectral mean* |
| Relative $\gamma$ power band | $\delta/\left( \alpha+\beta\right)$ power ratio | Spectral variance* |
| Spectral entropy | $\theta/\left( \alpha+\beta\right)$ power ratio | Spectral skewness |
| Renyi entropy | $\delta/\left( \alpha+\beta+\theta\right)$ power ratio | Spectral kurtosis |

##

## **SUPPLEMENTARY FIGURES**

**Figure S1.** Raw in-ear-EEG 30-second data sample for each subject.

**Figure S2.** Pre-processed in-ear-EEG 30-second data sample for each subject.

**Figure S3.** Color-coded heatmap showing the selection frequency of each extracted feature across the various subsets, separately for each sleep stage, reporting warmer colors for higher frequencies.

**Figure S4.** Head topography plots of JSD-FSI similarity-scores of the in-ear-EEG with respect to the PSG channels in the set (EEG and EOG channels) - for each subject in the awake stage. Unipolar channels are represented as markers; bipolar channels are represented as lines. The mean and the standard deviation of the similarity-scores distribution computed for each subject are also included. No JSD-FSI similarity-scores are reported for the channel M2 of subjects 3 and 6.

**Figure S5.** Head topography plots of JSD-FSI similarity-scores of the in-ear-EEG with respect to the PSG channels in the set (EEG and EOG channels) - for each subject in the NREM stage. Unipolar channels are represented as markers; bipolar channels are represented as lines. The mean and the standard deviation of the similarity-scores distribution computed for each subject are also included. No JSD-FSI similarity-scores are reported for the channel M2 of subjects 3 and 6.

**Figure S6.** Head topography plots of JSD-FSI similarity-scores of the in-ear-EEG with respect to the PSG channels in the set (EEG and EOG channels) - for each subject in the REM sleep stage. Unipolar channels are represented as markers; bipolar channels are represented as lines. The mean and the standard deviation of the similarity-scores distribution computed for each subject are also included. No JSD-FSI similarity-scores are reported for subjects 3 and 8 nor for the channel M2 of subject 6.

**Figure S7.** JSD-FSI similarity-scores distributions, i.e., distributions derived from Scalp-EEG-to-Scalp-EEG (blue line), and Scalp-EEG-to-In-ear-EEG (orange line) comparisons - for each subject in the awake stage. The area under the Scalp-EEG-to-In-ear-EEG distribution - related to JSD-FSI values greater than the minimum Scalp-EEG-to-Scalp-EEG score - is highlighted in purple.

**Figure S8.** JSD-FSI similarity-scores distributions, i.e., distributions derived from Scalp-EEG-to-Scalp-EEG (blue line), and Scalp-EEG-to-In-ear-EEG (orange line) comparisons - for each subject in the NREM stage. The area under the Scalp-EEG-to-In-ear-EEG distribution - related to JSD-FSI values greater than the minimum Scalp-EEG-to-Scalp-EEG score - is highlighted in purple.

**Figure S9.** JSD-FSI similarity-scores distributions, i.e., distributions derived from Scalp-EEG-to-Scalp-EEG (blue line), and Scalp-EEG-to-In-ear-EEG (orange line) comparisons - for each subject in the REM stage. The area under the Scalp-EEG-to-In-ear-EEG distribution - related to JSD-FSI values greater than the minimum Scalp-EEG-to-Scalp-EEG score - is highlighted in purple. No JSD-FSI similarity-scores are reported for subjects 3 and 8.

**Figure S10.** JSD-FSI similarity-scores distributions, i.e., distributions derived from EOG-to-EOG (green line), and EOG-to-In-ear-EEG (red line) comparisons - for each subject in the awake stage. The area under the EOG-to-In-ear-EEG distribution - related to JSD-FSI values greater than the minimum EOG-to-EOG score - is highlighted in purple.

**Figure S11.** JSD-FSI similarity-scores distributions, i.e., distributions derived from EOG-to-EOG (green line), and EOG-to-In-ear-EEG (red line) comparisons - for each subject in the NREM stage. The area under the EOG-to-In-ear-EEG distribution - related to JSD-FSI values greater than the minimum EOG-to-EOG score - is highlighted in purple.

**Figure S12.** JSD-FSI similarity-scores distributions, i.e., distributions derived from EOG-to-EOG (green line), and EOG-to-In-ear-EEG (red line) comparisons - for each subject in the REM stage. The area under the EOG-to-In-ear-EEG distribution - related to JSD-FSI values greater than the minimum EOG-to-EOG score - is highlighted in purple. No JSD-FSI similarity-scores are reported for subjects 3 and 8.
